# Functional hypoxia drives neuroplasticity and neurogenesis via brain erythropoietin

**DOI:** 10.1038/s41467-020-15041-1

**Published:** 2020-03-09

**Authors:** Debia Wakhloo, Franziska Scharkowski, Yasmina Curto, Umer Javed Butt, Vikas Bansal, Agnes A. Steixner-Kumar, Liane Wüstefeld, Ashish Rajput, Sahab Arinrad, Matthias R. Zillmann, Anna Seelbach, Imam Hassouna, Katharina Schneider, Abdul Qadir Ibrahim, Hauke B. Werner, Henrik Martens, Kamilla Miskowiak, Sonja M. Wojcik, Stefan Bonn, Juan Nacher, Klaus-Armin Nave, Hannelore Ehrenreich

**Affiliations:** 1 Clinical Neuroscience, Max Planck Institute of Experimental Medicine, Göttingen, Germany; 2 Neurobiology Unit, Program in Neurosciences and Interdisciplinary Research Structure for Biotechnology and Biomedicine (BIOTECMED), Universitat de València, Burjassot, Spain; 3 Institute of Medical Systems Biology, Center for Molecular Neurobiology, University Clinic Hamburg-Eppendorf, Hamburg, Germany; 4 Department of Neurogenetics, Max Planck Institute of Experimental Medicine, Göttingen, Germany; 5 Synaptic Systems GmbH, Göttingen, Germany; 6 Copenhagen Affective Disorder Research Centre, Psychiatric Centre Copenhagen, Copenhagen University Hospital, Rigshospitalet, Copenhagen, Denmark; 7 Department of Molecular Neurobiology, Max Planck Institute of Experimental Medicine, Göttingen, Germany; 8 DFG Research Center for Nanoscale Microscopy and Molecular Physiology of the Brain (CNMPB), Göttingen, Germany; 9 CIBERSAM: Spanish National Network for Research in Mental Health, Valencia, Spain; 10 Fundación Investigación Hospital Clínico de Valencia, INCLIVA, Valencia, Spain

**Keywords:** Cellular neuroscience, Cognitive neuroscience

## Abstract

Erythropoietin (EPO), named after its role in hematopoiesis, is also expressed in mammalian brain. In clinical settings, recombinant EPO treatment has revealed a remarkable improvement of cognition, but underlying mechanisms have remained obscure. Here, we show with a novel line of reporter mice that cognitive challenge induces local/endogenous hypoxia in hippocampal pyramidal neurons, hence enhancing expression of EPO and EPO receptor (EPOR). High-dose EPO administration, amplifying auto/paracrine EPO/EPOR signaling, prompts the emergence of new CA1 neurons and enhanced dendritic spine densities. Single-cell sequencing reveals rapid increase in newly differentiating neurons. Importantly, improved performance on complex running wheels after EPO is imitated by exposure to mild exogenous/inspiratory hypoxia. All these effects depend on neuronal expression of the *Epor* gene. This suggests a model of neuroplasticity in form of a fundamental regulatory circle, in which neuronal networks—challenged by cognitive tasks—drift into transient hypoxia, thereby triggering neuronal EPO/EPOR expression.

## Abstract (web summary)

EPO treatment improves cognition, but underlying mechanisms were unknown. Here the authors describe a regulatory loop in which brain networks challenged by cognitive tasks drift into functional hypoxia that drives—via neuronal EPO synthesis—neurodifferentiation and dendritic spine formation.

## Introduction

Erythropoietin (EPO) is a hypoxia-inducible growth factor in mammalian kidney, named after its role in hematopoiesis^1,2^. Unexpectedly, both EPO and its receptor (EPOR) were later detected in the brain, where they are upregulated by injury conditions. High-dose recombinant human (rh) EPO, a drug in clinical use for anemic patients, exerts neuroprotective and neuroregenerative effects that are independent of the hematocrit, which is mechanistically unexplained^3–8^. Moreover, rhEPO improves cognitive function and reduces gray matter loss in a range of neuropsychiatric conditions^9–13^. Even in healthy mice, rhEPO treatment improves cognition, which is associated with enhanced hippocampal long-term potentiation^14–16^. Surprisingly, rhEPO increases the number of mature hippocampal pyramidal neurons without underlying effect on cell proliferation or cell death^17^. This effect is mediated in neurons mainly by JAK-STAT, PI3K/AKT/PKB, Ras-MEK, and ERK1/2, as well as NF-κB; pathways widely comparable to the hematopoietic system^18–20^. This raises the question whether the expression of EPO and its receptor serves a physiological function in the nervous system, and what could be the triggering factors of EPO expression under physiological conditions.

## Results

### Generation of pyramidal neurons in adult mice and amplification by rhEPO

**Fig. 1:**
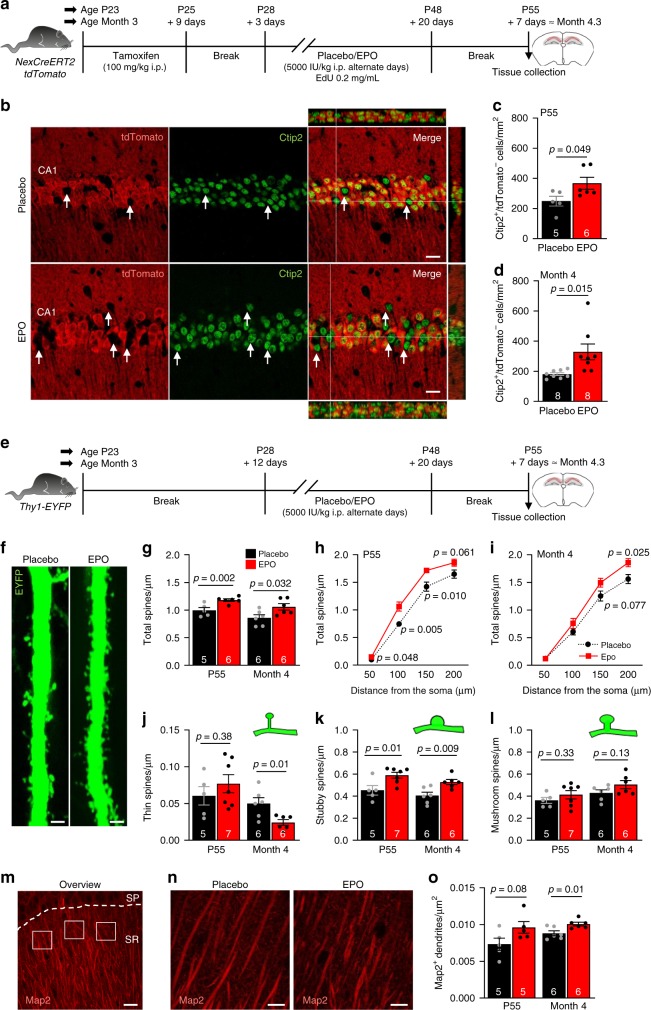
EPO increases number of pyramidal neurons and spine density in the CA1 region of the hippocampus. **a** Experimental design to determine the effect of EPO on neuron numbers: two cohorts of *NexCreERT2::tdTomato* mice (starting at age P23 or month 3) were administered tamoxifen (at P23: 5× i.p.; at month 3: 10× i.p.), followed by placebo or EPO (5000IU/kg; 11× i.p.) on alternate days for 3 weeks. **b** Representative images of tdTomato (red) and Ctip2 (green; excitatory neuronal marker) staining in CA1 of placebo or EPO-treated mice. White arrows indicate newly generated neurons. **c**, **d** Quantification of newly generated neurons (Ctip2^+^/tdTomato^−^) in CA1 of P55 **c** and ~4-month-old mice **d** treated with placebo or EPO. Data from *n* = 5/8 (placebo; 1c/d) and *n* = 6/8 (EPO; 1c/d) mice. **e** Experimental design to determine the effect of EPO on dendritic spine number and morphology: *Thy1-EYFP* mice (starting at age P28 or month 3) were treated with placebo or EPO (11× i.p.) on alternate days, for 3 weeks. **f** Representative images of EYFP (green) expression in principal apical dendrites in CA1 of placebo or EPO mice. **g**–**i** For P55 and ~4-month-old mice, total number of spines (per µm) are given as bar graphs **g** and, additionally, as line graphs **h**–**i** presenting their distance from the soma. Data from *n* = 5/6 (placebo; P55/4 months) and *n* = 6 (EPO) mice (6 cells/mouse analyzed). **j**–**l** Based on the morphology (illustrated as green insets), the number of thin **j**, stubby **k**, and mushroom **l** spines (per µm) were sub-quantified. **m** Representative image of the quantification method employed for determining the number of dendrites using dendritic marker Map2 (red) in CA1 stratum radiatum (SR); stratum pyramidale (SP). White boxes denote areas of measurements. **n** Representative images of Map2 (red) staining in ~4-month-old mice treated with placebo or EPO. **o** Quantification of Map2^+^ dendrites in P55 and 4-month-old mice treated with placebo or EPO. Data from *n* = 5/6 (placebo; P55/4 months) and *n* = 5/6 (EPO; P55/4 months) mice. Within bars, mouse *n* numbers indicated; mean ± SEM presented; two-tailed Student’s *t*-test; scale bars: **b** 50 µm; **f** 10 µm; **m** 20 µm; and **n** 10 µm. Source data underlying graphs **c**, **d**, **g**–**l**, and **o** are provided as a Source Data File.

First, we developed a method to directly label and quantify newly generated neurons in the hippocampal cornu ammonis (CA) field of adult mice. This was possible by permanently labeling all mature pyramidal neurons present at P27 using a tamoxifen-inducible reporter gene in *NexCreERT2::R26R-tdT* mice (Fig. 1a, b)^21^. Thus, all neurons differentiating and maturing after termination of the tamoxifen-induced Cre recombination lack tdTomato, but can be positively identified by Ctip2, a specific marker of pyramidal neurons, thereby revealing adult ‘neurogenesis’ independent of DNA synthesis.

Immediately after tamoxifen induction, at P28, the number of such unlabeled (tdTomato^−^, Ctip2^+^) pyramidal neurons, quantified for control purposes, was <3% of all Ctip2^+^ cells. When rhEPO treatment was initiated at P28 as described^17^, and mice were analyzed at P55, we found a considerable number of newly differentiated (tdTomato^−^/Ctip^+^) neurons in CA1, without evidence by 5-Ethynyl-2′-deoxyuridine (EdU) incorporation of proliferating precursors, as we reported previously^17^. Upon rhEPO treatment, the number of pyramidal cells was higher compared to placebo, but even untreated mice revealed a remarkable increase in new neurons (Fig. 1c).

Strikingly, when we applied the same rhEPO treatment to older mice (age 3 months), we detected a comparable effect on pyramidal neuron numbers (Fig. 1d). Thus, there is a substantial generation of pyramidal neurons from pre-existing (non-proliferating) precursors also in adulthood, suggesting a previously overlooked aspect of adult neurogenesis, discovered by serendipity in rhEPO-treated mice.

### EPO increases dendritic spine density

Using *Thy1-EYFP* transgenic mice^22^, we noted that EPO treatment also enhanced the dendritic spine density in pyramidal neurons at both P55 and 4 months (Fig. 1e–i). The analysis of spine morphology (Fig. 1j–l) showed an increase in stubby immature spines and a trend toward more mature mushroom spines^23^. Reflecting the increased pyramidal neuron number, we also noted a higher density of Map2 immunoreactive primary dendrites in the stratum radiatum (Fig. 1m–o).

### Immediate differentiation response of neuronal precursors to rhEPO

**Fig. 2:**
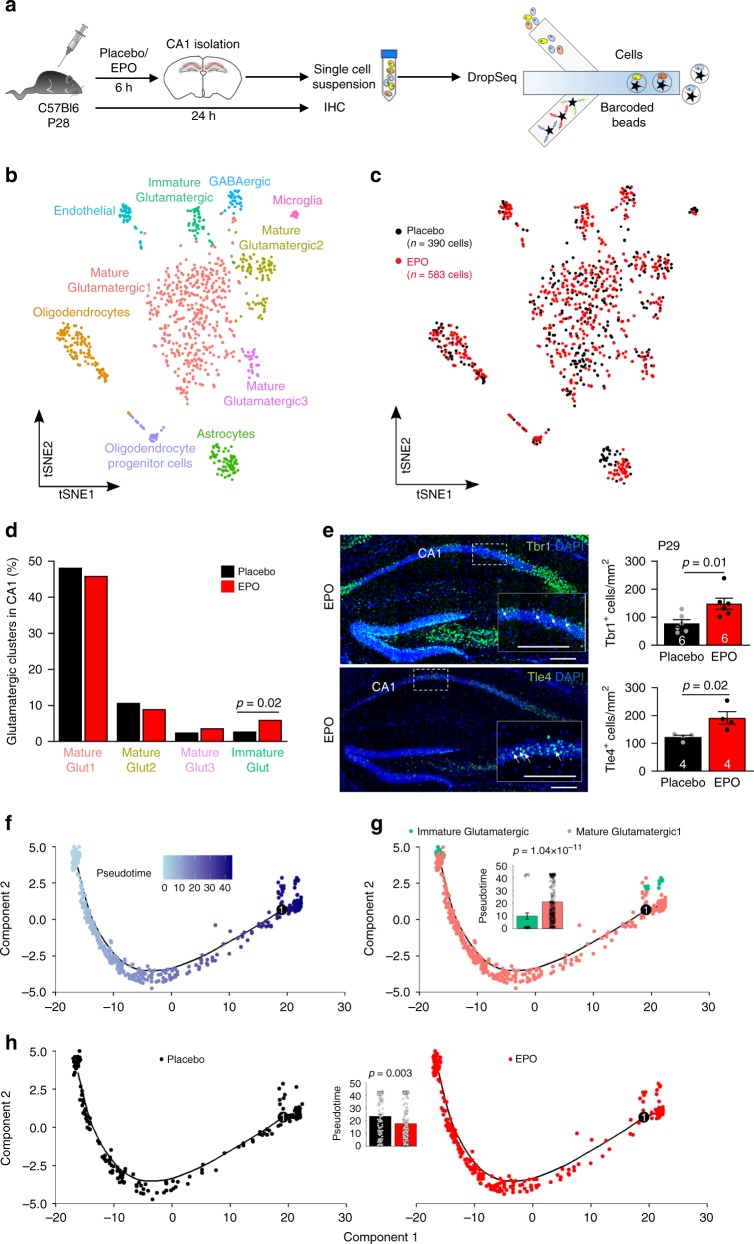
Transcriptome analysis (scRNA-seq) demonstrates an immediate increase in neurodifferentiation upon EPO application. **a** Experimental design of WT C57Bl6 mice (*n* = 3 per group), treated at P28 with a single i.p. injection of placebo or EPO (5000IU/kg), followed by isolation of the CA1 region after 6 h and processing for DropSeq analysis (processing of biological replicates performed separately, followed by pooling for graphical presentation and final analysis), as well as after 24 h for immunohistochemistry (figure created by Debia Wakhloo). **b** Visualization of hippocampal CA1 cell clusters using *t*-distributed stochastic neighbor embedding (*t*SNE). Each color represents a cluster of specific cells, characterized by a defined gene expression profile. **c** Individual cells derived from either EPO (red) or placebo (black)-treated mice are denoted. **d** Percentage of cells in the respective glutamatergic clusters per treatment condition (EPO: *n* = 583, placebo: *n* = 390); two-tailed Fisher’s exact test. **e** Representative images and quantification of Tbr1 and Tle4 staining (immature neuronal markers) in CA1 of mice at P29, i.e., 24 h after a single injection of EPO. Within bars, mouse n numbers indicated; mean ± SEM presented; two-tailed student’s *t*-test. **f** Trajectory analysis in *Monocle2* of cells in the ‘Mature Glutamatergic1’ and the ‘Immature Glutamatergic’ cluster colored by pseudotime (the darker the more mature). **g** Trajectory colored by cell identity. Bar graph indicates average pseudotime of the respective clusters; mean ± SEM; two-tailed Mann–Whitney *U* test. **h** Trajectory colored and split by placebo (black; left) versus EPO treatment (red; right). Bar graph shows average pseudotime of cells in the respective treatment groups; two-tailed Mann–Whitney *U* test; mean ± SEM; see also Supplementary Figs. 1–3. Source data underlying graph **e** are provided as a Source Data File.

We employed single-cell transcriptome analysis (scRNA-seq) to explore the immediate response of cells in CA1 to rhEPO, detectable 6 h after a single intraperitoneal (i.p.) injection of 5000U rhEPO/kg (Fig. 2, Supplementary Figs. 1–3, Source Data File). This revealed alterations in the cell cluster composition as defined by gene expression profiles^24^, with more cells in the immature glutamatergic differentiation cluster after rhEPO (Fig. 2a–d). Markers that characterize this cluster include doublecortin (*Dcx*), transducin-like enhancer family member4 (*Tle4*), T-box brain1 (*Tbr1*), rhabdomyosarcoma 2-associated transcript (*Rmst*), SRY-Box5 (*Sox5*), DNA-binding protein SATB1 (*Satb1*), ELAV-like neuron-specific RNA-binding protein4 (*Elavl4*), fucosyltransferase9 (*Fut9*), CUGBP Elav-like family member4 (*Celf4*), and heparan sulfate-glucosamine 3-sulfotransferase4 (*Hs3st4*). For two of them, *Tle4* and *Tbr1*, in situ validation of protein expression by immunohistochemistry (IHC), predominantly in Ctip^+^ neurons (confirmed by double labeling) at P29, i.e., 24 h after a single injection of EPO/placebo, exemplifies the distinct EPO effect (Fig. 2e). Note that Tbr1^+^ neurons upon EPO are rapidly and strongly detected in CA1 at P29, but only rarely anymore at P55. Tbr1^+^ cells in dentate gyrus serve as positive control for both time points (Supplementary Fig. 3e). Other neuronal clusters and non-neuronal cell types were not obviously different between treatment groups at 6 h (Supplementary Fig. 3b).

In order to further investigate the EPO-induced effects on cell differentiation, we performed a trajectory (pseudotime) analysis on cells in the immature glutamatergic cluster and its neighboring cluster, mature glutamatergic1, in *Monocle2*^25^; Fig. 2f; list of differentially expressed genes between these two clusters given in Source Data File). The analysis confirmed the immature identity of cells in the immature glutamatergic cluster, reflected by a homogeneously low pseudotime of almost all cells in this cluster, which was significantly lower as compared to cells in the mature glutamatergic1 cluster (Fig. 2g). After post hoc exploratory removal of immature cells with high pseudotime (>15, *n* = 10), the increase in immature cells upon EPO still remained significant (*p* = 0.039; Fisher’s exact test, one sided). Even if the immature cells with high pseudotime (>15) were reassigned to the mature glutamatergic cluster, a significance level of *p* = 0.041 was obtained. Importantly, we observed a generally lower pseudotime upon EPO, independent of cluster assignment, further supporting our observation of an increase in immature cells and excluding a mere ‘clustering artifact’ (Fig. 2h). Also, reclustering in *Monocle2* of cells from the two clusters revealed a robust detection of the immature cluster across methods (*Seurat* and *Monocle2*; Supplementary Fig. 3a) and a robustly increased number of immature cells upon EPO as compared to placebo treatment, independent of the analysis platform (*p* = 0.0008). For further validation of the immature identity of these cells see Supplementary Fig. 3c, d.

Together, these data provide supportive evidence of an immediate effect of EPO on pre-existing precursors to instantaneously initiate neuronal differentiation, thereby rapidly increasing the numbers of cells in the immature glutamatergic differentiating cluster. Expectedly, EPO and EPOR were not detected by scRNA-seq due to their very low expression level, a known drop-out effect in scRNA-seq data.

### Elevated expression of EPO and EPOR by pyramidal neurons upon CRW

**Fig. 3:**
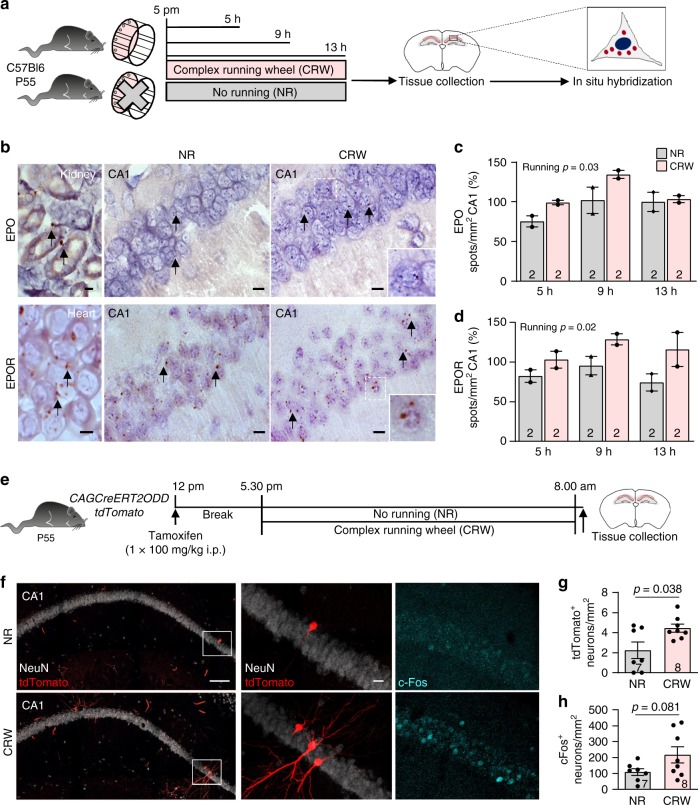
Expression of the EPO/EPOR system in pyramidal neurons shown by ISH and its upregulation by functional hypoxia. **a** Experimental design of WT C57Bl6 mice exposed to no running (NR) or voluntary running on CRW at P55 for 5, 9, or 13 h. ISH was performed on CA1 regions (figure created by Debia Wakhloo). **b** Representative ISH images of CA1 regions of non-runners (NR) and runners (CRW), demonstrating EPO and EPOR mRNA expression in pyramidal neurons (brown spots). As positive controls for EPO/EPOR mRNA expression, sections of kidney (P55) and heart (E14.5) from WT C57Bl6 mice are presented. **c**, **d** Quantification of total number of EPO or EPOR mRNA spots in CA1 of non-runners (NR) and runners (CRW), shown at 5, 9, and 13 h; mean and range shown; two-way ANOVA; *n* numbers indicated within bars; scale bars in **b**: 10 µm; for heart 5 µm. **e** Experimental design to determine the effect of running on the induction of functional hypoxia in the CA1 region. *CAGCreERT2-ODD::tdTomato* mice were administered tamoxifen at P55 of age and exposed to either no running (NR) or overnight voluntary CRW. **f** Representative images of neuronal marker NeuN (white), tdTomato (red; labeling hypoxia), and cFos (cyan) in non-runner (NR) and runner mice (CRW); scale bars 100 µm (left); 10 µm for magnifications. **g**, **h** Quantification of hypoxic neurons (tdTomato^+^) and active neurons (cFos^+^) in CA1 after CRW versus NR overnight (12 h); mean ± SEM; two-tailed Student’s *t*-test; data from *n* = 7 (non-runners, NR) and *n* = 8 (CRW) mice. Source data underlying graphs **c**, **d**, **g**, and **h** are provided as a Source Data File.

Searching for the physiological significance of these responses, we explored whether EPO and EPOR are endogenously expressed by pyramidal neurons. Using a highly sensitive in situ hybridization (ISH) method, capable of detecting even single mRNA molecules, we identified both EPO and EPOR transcripts in pyramidal neurons of the hippocampal CA1 region (Fig. 3a–d). The overall mRNA abundance in the hippocampus was higher than in cortex and other brain regions. Interestingly, when mice were placed into cages with complex running wheels (CRW)^26^, a cognitive challenge that stimulates intricate motor learning and coordination, only 5–9 h of running in the dark phase were sufficient to upregulate the expression of EPO and EPOR transcripts in pyramidal neurons (Fig. 3a–d).

### Permanent labeling of hypoxic neurons in CA1 by CRW exposure

We therefore wondered which mechanism would lead to the swift upregulation of EPO in pyramidal neurons upon cognitive challenge. In the kidney, it is hypoxia and hypoxia-inducible factors (Hif), which are well known to induce EPO expression^1,2,7,27^. We therefore hypothesized that cognitive challenge, such as learning of complex motor tasks, is associated with neuronal activity that requires more oxygen than present in the normal steady state. This could induce a mild (transient) endogenous hypoxia as a potential signal underlying upregulated expression of EPO in highly active pyramidal neurons. To test this hypothesis, we asked whether ‘functional hypoxia’ can be detected in the brain after cognitive challenge, such as complex wheel running. To this end, we created a novel transgenic mouse for the ubiquitous expression of a chimeric fusion protein between the tamoxifen-inducible CreERT2 recombinase and the oxygen-dependent degradation (ODD) domain of Hif-1α^28^. In mice bearing this *CreERT2-ODD* transgene, driven by the CAG promoter, ‘fate mapping’ and identification of (transiently) hypoxic cells is possible with the tdTomato reporter (*CAGCreERT2-ODD::R26R-tdTomato*). Indeed, exposing these mice after tamoxifen injection to CRW over one night, we determined a distinctly increased number of ‘ODD-labeled’ pyramidal neurons in the CA1 region when compared to non-runners. This indicates that a new challenging task has led to more (transiently) hypoxic neurons, which became permanently labeled. Parallel quantification of cFos expression as neuronal activity marker^29^ revealed a tendency of increased cFos labeling upon CRW (Fig. 3e–h), but no double labeling with ODD-tdTomato. The latter likely occurs too late in a respective cell, i.e., when its immediate early gene (cFos) expression has already disappeared. In contrast, after only 4 h of CRW, cFos labeling was obvious, but no ODD-tdTomato labeling yet detected (cFos^+^ cells: non-runners 40.1 ± 20.8, *n* = 3; CRW 143.3 ± 24.4, *n* = 7; *p* = 0.034, mean ± SEM). Additional confirmation of hypoxic cell labeling by ODD-tdTomato was obtained using pimonidazole, resulting in findings similar to a previous report on heart^28^ (Supplementary Fig. 4).

### EPO treatment strongly increases motor learning and endurance

To further address the potential of rhEPO to lastingly improve cognitive performance, we treated juvenile mice with our protocol that results in ~20% more pyramidal neurons (starting at P28, i.p. injection of rhEPO/placebo every other day for 3 weeks, followed by a 1-week break). Afterward, the running-naive mice were transferred to cages containing CRW. During the initial learning phase, a rise in performance was observed, clearly dissociating EPO from placebo-treated mice after just a few hours, followed by enhanced endurance over the whole night.

### Deletion of neuronal EPOR attenuates EPO-/hypoxia-induced motor learning

**Fig. 4:**
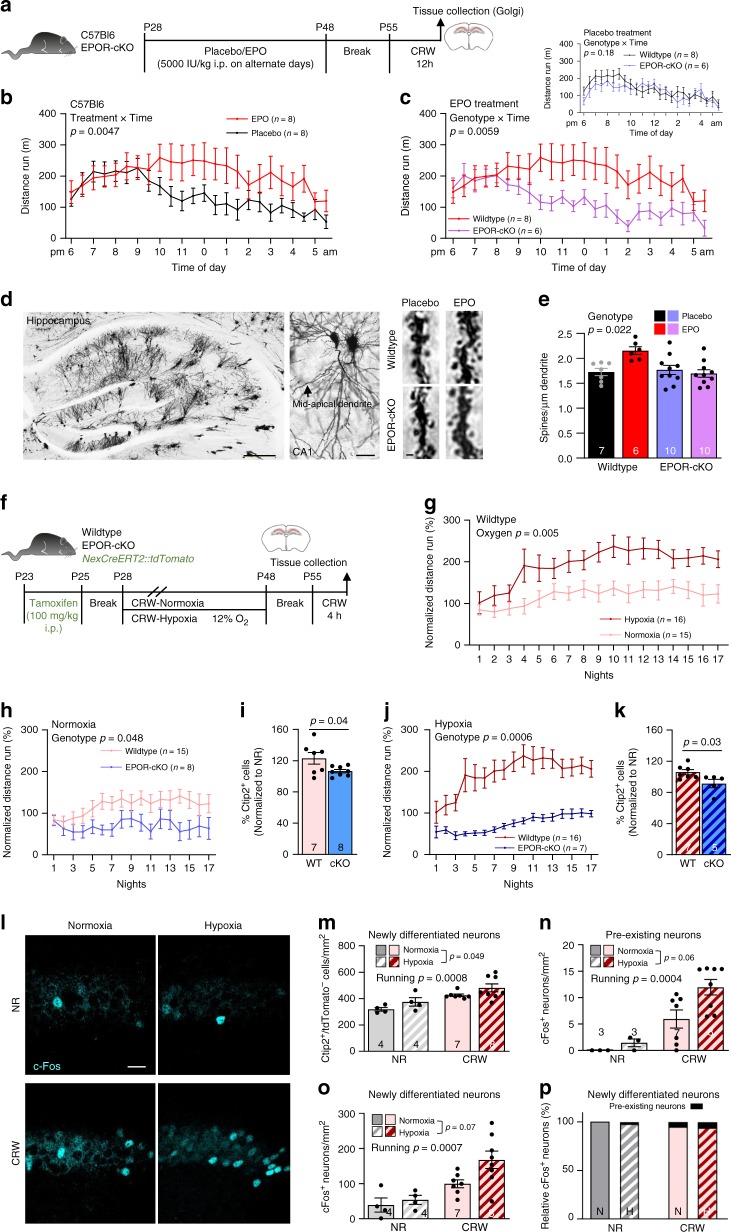
Targeted deletion of EPOR in pyramidal neurons attenuates EPO/hypoxia-induced motor learning. **a** Experimental design. **b** Total distance run by placebo (black) and EPO (red) treated WT mice. **c** Total distance run by WT (red) and EPOR-cKO mice (purple) following EPO; insert shows for comparison total distance run by WT (black) and EPOR-cKO mice (blue) upon placebo. **d** Representative images of hippocampal section processed with GolgiStain^TM^Kit (scale bar: 100 µm), CA1 pyramidal neurons (scale bar: 20 µm), and mid-apical dendrites (scale bar: 1 µm). **e** Quantification of dendritic spines in WT and EPOR-cKO mice treated with placebo or EPO. **f** Experimental design. WT and EPOR-cKO mice were exposed to NR or CRW under normoxia (21% O_2_) or inspiratory hypoxia (12% O_2_), starting at P28 for 3 weeks. After 1 week of break, CRW mice were again exposed to voluntary CRW for 4 h (cFos activation) before being sacrificed at P55 (results in **g**–**k**); *NexCreERT2::tdTomato* mice were injected with tamoxifen (5× i.p.—starting at P23) and exposed to same experimental paradigm (results in **l**–**p**). **g** CRW curves over 17 nights (normalized to mean distance over first three nights) of WT mice exposed to continuous inspiratory normoxia versus hypoxia. **h**–**k** Effect of EPOR deletion in pyramidal neurons: **h** CRW curves of WT and EPOR-cKO mice exposed to normoxia and **i** quantification of their Ctip2^+^ cells in CA1 (normalized to NR controls). **j** CRW curves of WT and EPOR-cKO mice exposed to inspiratory hypoxia, and **k** quantification of their Ctip2^+^ cells in CA1 (normalized to NR controls). **l** Representative images of cFos^+^ neurons (cyan) in NR and CRW mice exposed to normoxia or hypoxia (scale bar: 25 µm). **m** Quantification of newly formed neurons (Ctip2^+^/tdTomato^−^) in NR and CRW mice exposed to normoxia or hypoxia. **n**, **o** Quantification of active neurons classified as pre-existing neurons (cFos^+^/Ctip2^+^/tdTomato^+^) or as newly formed neurons (cFos^+^/Ctip2^+^/tdTomato^−^) in CA1 of mice quantified in **m**. **p** Presentation of the small percentage of pre-existing neurons among cFos^+^ neurons. Data given as mean ± SEM; two-tailed Student’s *t*-test (i & k) or two-way ANOVA; mouse *n* numbers in panels. Source data of graphs **b**, **c**, **e**, **g**–**k**, and **m**–**p** provided as Source Data File.

Theoretically, the EPO-induced increase in hematocrit could account for this enhanced performance. To rule this out and to explore whether pyramidal EPOR is mediating this effect, we deleted the *Epor* gene from pyramidal neurons (*NexCre::EpoRfl/fl*; Supplementary Fig. 5). More details on the generation of this conditional *Epor* allele, its recombination with different Cre-lines, and a comprehensive phenotype analysis will be described elsewhere (manuscript in preparation). As delineated in Fig. 4a–c, the remarkable rise in performance of wild-type (WT) mice following EPO treatment reported above was well replicated, whereas EPOR-cKO mice failed to show any noticeable effect of EPO treatment on CRW performance (i.e., complex motor learning and endurance). Placebo-treated EPOR-cKO did not appreciably differ from WT mice (Fig. 4c, inset). The behavioral phenotype of EPOR-cKO mice strongly suggested that also neuronal complexity, i.e., dendritic spine density of pyramidal neurons, would be affected by EPOR deletion. Indeed, Golgi staining revealed that lack of EPOR in pyramidal neurons prevented the EPO-induced increase in dendritic spines (Fig. 4d, e).

### Mild inspiratory hypoxia acts synergistically with cognitive challenge

Since learning to run on CRW leads to endogenous (‘functional’) hypoxia in pyramidal neurons, as demonstrated by ODD labeling, and since pyramidal EPO and EPOR mRNAs are upregulated by functional hypoxia, we wondered whether CRW exposure for several weeks would (similar to rhEPO treatment) also lead to more pyramidal neurons. Moreover, would a sustained exposure of mice to a mild exogenous (inspiratory) hypoxia (12% O_2_), when coinciding with running activity, result in similar or even synergistic effects, i.e., better learning performance and more neurons in the stratum pyramidale? In the respective large-scale, multi-arm experiment over 3 weeks, we indeed detected improved motor learning and endurance on the CRW of mice under mild hypoxia when compared to normoxia (Fig. 4f–k). To unequivocally demonstrate that the neuronal EPO/EPOR system is responsible for the observed effects, we employed again our mice with targeted deletion of the *Epor* gene in pyramidal neurons. In fact, lack of EPOR in pyramidal neurons resulted in a distinct reduction of the learning curve slope over time, both under normoxic and hypoxic conditions, when compared to the respective WT controls. Also, non-running EPOR-cKO mice have already less pyramidal neurons independent of hypoxia (normoxia: 7834.07 ± 223.18 in cKO versus 9377.48 ± 864.48 in WT; hypoxia: 8431.51 ± 320.40 in cKO versus 10730.78 ± 497.00 in WT; overall genotype effect *p* = 0.0012) and fail to show the increase in pyramidal neurons found in WT mice upon running with or without hypoxia (Fig. 4f–k).

### Expression of cFos marks newly differentiated neurons

Under both normoxic and hypoxic conditions, we counted more recently differentiated (EdU negative) pyramidal neurons in runners than in non-runners in the hippocampal CA1 field. We thus wondered whether the newly differentiated pyramidal neurons, generated in mice placed into CRW cages, are functional. To address this question, we challenged animals for 3 weeks in CRW (with or without mild inspiratory hypoxia) but moved them first back to regular cages for 1 week, in analogy to the rhEPO treatment scheme. Then, we re-exposed the mice to CRW for only 4 h at the beginning of their dark phase. Immediately thereafter, they were sacrificed and brains processed for IHC. As readout of neuronal activity and functional integration, cFos expression^29^ was used again and found to be enhanced following hypoxia, and in particular after running. Unexpectedly, cFos was mainly detected in the recently differentiated neurons, whereas the fraction of pre-existing neurons expressing cFos was small (Fig. 4l–p).

## Discussion

**Fig. 5:**
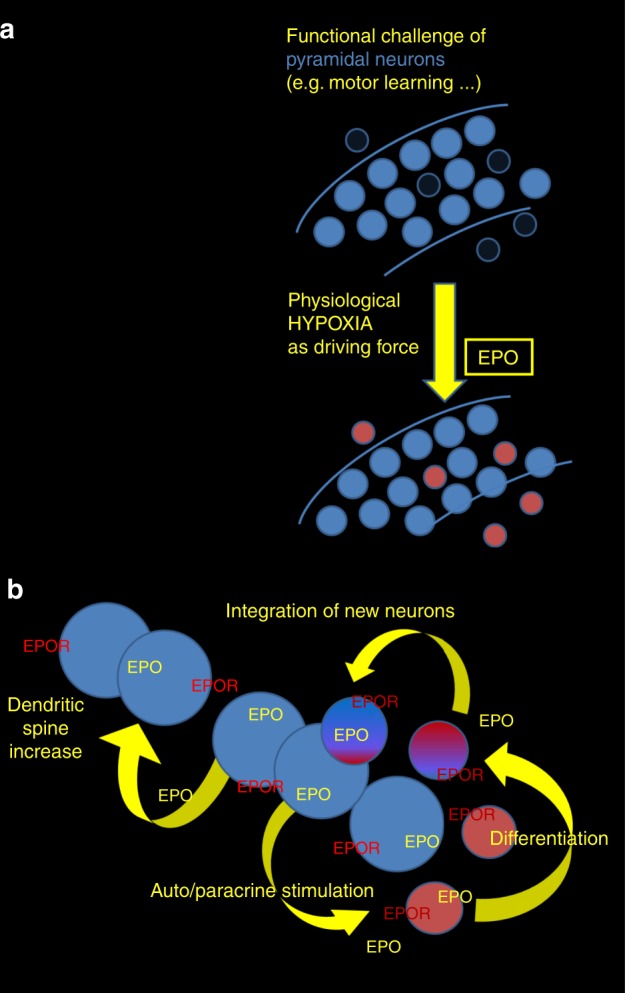
Comprehensive working model of EPO function in the brain. Panels illustrate the suggested cellular mechanisms and effects of EPO/EPOR in CA1 (hypotheses and respective figure created by Hannelore Ehrenreich). **a** Functional challenge of pyramidal neurons (blue) via, e.g., learning of new complex tasks provokes physiological hypoxia of the involved neurons. Hypoxia in turn induces neuronal production/release of EPO, which initiates differentiation of diverse non-proliferating precursors (red) with currently unknown identity to neurons. **b** Neuronal EPO binds in an auto/paracrine manner to EPOR on pyramidal neurons (blue), leading to an increase in dendritic spines. EPO simultaneously binds to EPOR on likely diverse neighboring cells (red), ready to differentiate into neurons on demand. Together, these integrative steps may explain the consistently found improvement of cognitive function under rhEPO treatment and, even more importantly, delineate physiological mechanisms, accomplishing lasting adaptation to challenge via the brain EPO system.

Taken together (Fig. 5), we have discovered that EPO - experimentally and clinically shown to improve cognition^9–17^ - stimulates a previously unrecognized mechanism of cellular neuroplasticity. Specifically, rhEPO (when peripherally administered) enters the brain, where it mimics the effects of endogenous neuronal EPO/EPOR signaling, stimulating dendritic spine formation on mature cells, and the differentiation of new pyramidal neurons from pre-existing (non-proliferating) precursors, the identity of which is currently unknown.

The EPO-responsive cells undergoing adult neurogenesis likely have no defining single genetic marker, suitable for CreER-based lineage tracing (unlike the EPO-responsive OPC^17^). Not even direct transdifferentiation of endothelial and astroglial cells or dedifferentiation of neurons can be completely excluded at this point. Jak2-Stat5 signaling, however, a pathway important for neuronal EPOR effects^18,20^, is known in various tissues to induce terminal differentiation, not dedifferentiation.

We hypothesize that the entire precursor cell lineage that is ready to differentiate toward pyramidal neurons in adult mice remains ‘in flow’. Similar to adaptive metabolic pathways, in which the ‘flux’ of metabolites matters more than the different steady-state concentrations, also in the proposed neuronal lineage progression, the EPO-responsive progenitor cells may never constitute abundant clusters in a cross-sectional steady-state analysis. This is remarkably similar to the effects of EPO on multiple precursors at different stages in the hematopoietic system^1,30–32^.

Pyramidal neurons, when challenged by novel tasks, undergo mild hypoxia, as detected here by ‘ODD labeling’, using hypoxia reporter mice. This activity-induced ‘functional hypoxia’ stimulates endogenous EPO expression by pyramidal neurons. In parallel, we find also EPOR expression enhanced, caused either directly by hypoxia^33^ or by EPO in an auto/paracrine manner^34^. When EPOR expression is deleted from pyramidal neurons, the endogenous EPO/EPOR system can no longer contribute to adaptive increase in performance.

Contrary to popular viewpoints that hypoxia is detrimental, recent reviews also consider beneficial effects and protection against cognitive dysfunction^35,36^. Our data using CRW indicate that hypoxia can act as driving force of long-lasting cellular neuroplasticity, complementing synaptic plasticity. Neuronally produced EPO/EPOR likely constitutes an auto/paracrine mechanism, possibly in concert with other hypoxia-inducible genes, like vascular endothelial growth factor, which has previously also been reported to enhance cognition^37^. We suggest a model of an intrinsic EPO/EPOR-mediated mechanism of cellular neuroplasticity (Fig. 5), in form of a fundamental regulatory circle, which can explain the remarkable procognitive effects of rhEPO, consistently found in humans and rodents, health and disease^9,10,12,14–16,38^.

A recent study documented EPO mRNA in different neural cell types in the embryonic brain (mouse organogenesis cell atlas—MOCA^39^). The extraordinarily low expression of EPO and EPOR in the adult brain may explain why this system has previously not been rigorously studied. So far, we have focused on the hippocampal CA1 region, where we had obtained first evidence of substantially increased neuron numbers after rhEPO treatment^17^. CA1 is involved in temporal pattern association and different facets of memory formation and consolidation^40^. Future studies will have to address EPO/EPOR functions in other CNS regions, including cortical areas known to be also engaged in CRW learning^41^, as well as neuron–glial interactions.

‘Adult neurogenesis’ from pre-existing precursors without proliferation extends the concept and pivotal work from many groups who discovered adult neurogenesis in distinct brain regions based on the selective labeling of proliferating cells in S-phase^42–48^. More complex than oligodendrogenesis from pre-existing precursors^49^, multiple neuronal progenitors may be relevant for maintaining neuron numbers in steady state, for adjustment to demand, and for rapid regenerative processes. The genetic approach of EPOR deletion in pyramidal neurons, together with the discovery of ‘functional hypoxia’ in the behaving brain, allowed us to propose a novel working model (Fig. 5). This model may add to our present concepts of neuroplasticity and adult neurogenesis.

## Methods

### Experimental models and mouse genetics

All experiments were approved by and conducted in accordance with the regulations of the local Animal Care and Use Committee (Niedersächsisches Landesamt für Verbraucherschutz und Lebensmittelsicherheit, LAVES). WT C57BL/6N (Charles River), *NexCreERT2*^21^, *R26R-tdTomato*^50^, *Thy1-YFPH* (Jackson Laboratory, 003782), *CAGCreERT2-ODD*^28^, *NexCre*^51^, and EPOR-floxed (EPOR^fl/fl^) mice were used for the experiments. Juvenile (P23) and adult (3 months old) mice were used in this study. For genetic labeling of projection neurons, *NexCreERT2* mice were bred with *Rosa26 floxed-stop tdTomato (R26R-tdTomato)* reporter mice to generate *NexCreERT2:R26R-tdTomato* mice. For labeling cells undergoing hypoxia, *CAGCreERT2-ODD* mice were employed^28^. Briefly, a fusion protein consisting of the ODD domain of Hif-1α and a ubiquitous *CAGCreERT2* is expressed upon tamoxifen induction. *CAGCreERT2-ODD* were bred with *R26R-tdTom*ato to generate *CAGCreERT2-ODD:tdTomato* reporter mice. Upon tamoxifen induction, cells are irreversibly labeled with tdTomato containing stabilized Hif-1α. *NexCre::Epor*^*fl/fl*^ mice were generated to specifically delete *Epor* in projection neurons. For the generation of *Epor*^*fl/fl*^ mice, embryonic stem cells (ES) harboring an engineered allele *(Epor*^*tm1a(KOMP)Wtsi*^*)* of the *Epor* gene were acquired from the Knockout Mouse Project (KOMP, University of California, Davis CA 95618, USA). ES cells were microinjected into blastocysts derived from C57BL/6N mice and the embryos were transferred to pseudopregnant foster mothers, yielding chimeric males. For ES clone *EPD0316_5_A03*, germline transmission was achieved upon breeding with C57BL/6N females, generating mice harboring the *Epor*^*tm1a(KOMP)Wtsi*^ allele (termed *Epor*^*lacZ-neo*^). The lacZ-neo cassette was excised in vivo upon interbreeding with mice expressing FLIP recombinase (*129S4/SvJaeSor-Gt(ROSA)26Sor*^*tm1(FLP1)Dym/J*^; backcrossed into C57BL/6N), yielding mice carrying the *Epor*^*tm1c(KOMP)Wtsi*^ allele (termed *Epor*^*flox*^). To recombine the *Epor* gene specifically in projection neurons, exons 3–6 were excised in vivo upon appropriate interbreedings of *Epor*^*tm1c(KOMP)Wtsi*^ mice with mice expressing Cre recombinase under control of the *Nex1/Neurod6* promoter^51^, generating mice carrying the *Epor*^*tm1d(KOMP)Wtsi*^ allele (*NexCre::Epor*^*fl/fl*^ mice).

All mice were housed in a temperature controlled environment (21 ± 2 °C) on a 12 h light–dark cycle with food and water available ad libitum. Male mice of the same age were used for the experiments, unless stated otherwise. All animals were genotyped before the start of each experiment. Detailed PCR protocols are available on request.

### Mouse treatment

*Tamoxifen:* Tamoxifen solution (10 mg/ml) was freshly prepared by dissolving tamoxifen freebase (Sigma) in corn oil (Sigma) at room temperature (RT) for 45 min. Postnatal CreERT2 activity in *NexCreERT2* mice was induced by a total of five i.p. injection of 100 mg/kg tamoxifen over the course of 3 days in juvenile mice. For CreERT2 induction in adult *NexCreERT2* mice, a total of ten i.p. injections of 100 mg/kg tamoxifen were administered over the course of ten consecutive days. For the desired induction of CreERT2 in *CAGCreERT2-ODD* mice, a single i.p. injection of 100 mg/kg tamoxifen was sufficient.

*EPO:* Male mice were i.p. injected with 5000IU/kg rhEPO (NeoRecormon, Roche) or placebo (solvent solution, 0.01 ml/g). At 48 h after the last tamoxifen injection, EPO/placebo treatment was initiated in P28 or 3-month-old *NexCreERT2, Thy1-YFPH* and also in P28 old WT and *NexCre::EPOR*^*fl/fl*^ mice, and carried out every other day for 3 weeks. For DropSeq analysis, EPO was administered once followed by tissue collection 6 h later. Additionally, for labeling of proliferating cells, *NexCreERT2* mice obtained EdU (0.2 mg/ml; ThermoFisher) via drinking water (exchanged on all alternate days).

*Hypoxia:* The hypoxia chamber was designed in cooperation with Coy Laboratory Products Inc. (Grass Lake, MI, USA) with the dimensions 164 cm × 121 cm × 112 cm. The system includes an air filtration system consisting of carbolime and activated charcoal. The oxygen and carbon dioxide levels were constantly detected and controlled via online monitoring. A gradual reduction of 3% oxygen per day resulted in 12% oxygen level in 3 days and was maintained until the end of the experiment. During the course of the experiment, mice were treated with EdU via drinking water as described above.

### Complex running wheels (CRW)

Post weaning, mice were single-housed and tamoxifen treatment was initiated for *NexCreERT2::tdTomato* mice (described above). *NexCreERT2::tdTomato* and *NexCre::EpoR*^*fl/fl*^ mice were divided into four groups and monitored for 17 days. Groups included (1) normoxic room conditions (at 21% O_2_) in standard cages, (2) normoxic conditions with voluntary running on CRW, (3) hypoxic conditions (hypoxia chamber to 12% O_2_) in standard cages, and (4) hypoxic conditions with voluntary running on CRW. CRW (TSE Systems, Bad Homburg, Germany) is characterized by randomized missing bars as previously described^26,41^. The testing period of 17 days was followed by 1 week of normal conditions for all groups (no running, normoxia). The mice that were previously running (in normoxic or hypoxic conditions) were finally exposed again to CRW for 4 h (as cFos inducing challenge) before being sacrificed (Fig. 4f). For ISH experiments, male WT mice (P55) were exposed to 5, 9, or 13 h of CRW (Fig. 3a). For the experiment involving *CAGCreERT2-ODD::tdTomato* mice, animals were exposed to overnight complex wheel running (Fig. 3e). Running was voluntary at all times with ad libitum access to food and water. Control mice (no running) were housed in standard cages. Mice were sacrificed, perfused, and brains collected as described below. Running was tracked automatically via Phenomaster software (TSE Systems, Germany) for the whole day. Since mice are mainly night-active (dark phase), the total distance run between 6 p.m. and 6 a.m. was summarized for every individual animal. The total distance run on each night was normalized to the average distance of each animal run in the first 3 nights (data expressed as % performance in relation to the first 3 nights). For the experiments involving EPO treatment, mice were treated with EPO (5000IU/kg, 11 i.p. injections on alternate days for 3 weeks), followed by 1-week break. They were then exposed to 12 h of CRW overnight (Fig. 4a–c). Data for this experiment are expressed as distance run summarized for every 30 min.

### Immunohistochemistry (IHC)

Mice were anesthetized and perfused transcardially with 4% cold formaldehyde. Dissected brains were post fixed in 4% formaldehyde at 4 °C and equilibrated in 30% sucrose dissolved in phosphate-buffered saline (PBS) at 4 °C overnight. Brains were then embedded in cryoprotectant (O.C.T.^TM^ Tissue-Tek, Sakura) and stored at −80 °C. Whole mouse brains were cut into 30 μm thick coronal sections (coordinates from bregma: −1.34 to −2.54 mm posterior) using a cryostat (Leica) and stored in a cryoprotective solution (25% ethylene glycol and 25% glycerol in PBS) at −20 °C until further use. For analysis of dendritic spines and Map2 dendrites, the right hemisphere, destined to the neuronal structural analysis, was cut in 100 μm coronal sections with a vibratome (Leica VT 1000E, Leica), collected in three subseries and stored at 4 °C in PB 0.1 M with sodium azide (0.05%).

For IHC, sections were permeabilized in PBS containing 0.3% Triton X-100 and blocked in 5% horse serum for 1 h at RT. Brain sections were incubated with primary antibodies in blocking solution overnight at 4 °C, followed by 2 h incubation of appropriate fluorophore-conjugated secondary antibodies in blocking solution containing 3% horse serum (or 5% normal donkey serum) and counterstained with 4′,6-diamidino-2-phenylindole (DAPI). The sections were then mounted on SuperFrostPlus Slides (ThermoFisher) with Aqua-Poly/mount (Polysciences, Inc). Primary antibodies used were: anti-Ctip2 (1:500; Guinea pig polyclonal; SYSY 325005), anti-Map2 (1:1000; mouse monoclonal; Sigma M9942), anti-Tbr1 (1:200; rabbit monoclonal; Abcam ab183032), anti-Tle4 (1:200; rabbit polyclonal; Abcam ab64833), anti-NeuN (1:500; mouse monoclonal; Millipore MAB377), and anti-cFos (1:1000; rabbit polyclonal; SYSY 226003). Secondary antibodies used were: Alexa488 anti-Guinea pig (1:500; Jackson Immuno Research 706-548-148) and Alexa635 anti-mouse (1:400; ThermoFisher A31575). Depending on the need for a triple or a quadruple staining, Alexa405 anti-rabbit (1:1000; Abcam 175652) or Alexa647 anti-rabbit (1:500; ThermoFisher A31573) were used. Following IHC, sections were stained for EdU using Click-iT^TM^ EdU Alexa Fluor^TM^ 647 Imaging kit (ThermoFisher E10415), according to the manufacturer’s instructions.

### Golgi–Cox staining

Mice were anesthetized and decapitated. The brain was removed, shortly rinsed in PBS, and processed with the ND Rapid GolgiStain^TM^ Kit (FDNeuroTechnologies, PK401) according to the manufacturer’s instructions. After impregnation, the brains were embedded in 2% Agar Agar in PBS and cut in 100 µm coronal sections with a vibratome (Leica, VT 1000 S) and placed on gelatin-coated glass slides (FDNeuroTechnologies, PO101). The slices were stained and dehydrated as described in the kit protocol. Slices were mounted with Perimount (ThermoFisher).

### Imaging and analysis

*Pyramidal neurons:* Imaging was performed using the Andor Eclipse TiE microscope system (Nikon, Tokyo, Japan) with a 40× objective (Plan Apo λ 40×, NA = 0.95) to image the hippocampal layers. Ctip2^+^ cells among total neuron numbers were manually counted. Ctip2^+^ and tdTomato^−^ cells were characterized as newly generated neurons. Quantifications are expressed as number of newly generated neurons divided by the total area of CA1 stratum pyramidale (mm^2^). For Ctip2 staining, a total of 16 hippocampi with 8 sections per animal were used. Quadruple (tdTomato, Ctip2, cFos, and EdU) staining was acquired with a TCS SP5-II System (Leica) equipped with a 20× objective (NA = 0.70). For total Ctip2^+^ counts, six hippocampi with three sections per animal were used. Quantifications for CRW mice were expressed as percentage of Ctip2^+^ cells normalized to their respective non-running controls. Whole hippocampi were imaged and analyzed by Fiji software. Neurons showing positive immunoreactivity for cFos were identified and quantified manually. These neurons were further sub-categorized into pre-existing (cFos^+^, tdTomato^+^, Ctip2^+^) or newly generated (cFos^+^, tdTomato^−^, Ctip2^+^) neurons. Again, quantifications are indicated as cFos^+^ cells divided by the total area of the CA1 stratum pyramidale (mm^2^). For cFos staining, a total of eight hippocampi with four sections per animal were used. For quantification of hypoxic neurons, whole hippocampus sections of *CAGCreERT2-ODD::tdTomato* mice were imaged. The tdTomato^+^ cells were characterized as neurons undergoing hypoxia and manually quantified. Quantifications are expressed as number of hypoxic neurons divided by the total area of CA1 stratum pyramidale (mm^2^). A total of ten hippocampi with five sections per animal were used. The cFos^+^ cells were also analyzed as described above. For Map2^+^ dendritic density analysis, the stratum radiatum of the CA1 region was imaged using a laser scanning confocal microscope (Leica TCS SPE). Per animal, three dorsal hippocampal slices were selected starting at bregma −1.58 mm. Confocal *z*-stacks (0.38 µm intervals) of whole sections were taken using a 63× objective (NA = 1.40). Per hippocampus, three images were captured, and four fields of the dimension 36.67 µm × 36.67 µm were analyzed in each image (total 36 fields per animal). Images were processed using Fiji software and Map2^+^ principal apical dendrites were manually quantified. The quantifications are expressed as number of Map2^+^ dendrites divided by the area of each image (1344.7 µm^2^).

*Dendritic spines:* Images were captured using a 63× oil immersion objective (NA = 1.40) and a 3.5× additional digital zoom to investigate the first 200 µm of the principal apical dendrite in segments of 50 µm. Confocal *z*-stacks (0.38 µm intervals) of whole sections were performed. Dendrites included in the study were at least 200 µm in length. Per animal, six such dendrites of six different *Thy1-YFPH* expressing pyramidal neurons were randomly selected from the CA1 region. The stitching plugin in Fiji software (2.0.0) was used to reconstruct three-dimensional images of the apical dendrites. The spines were further sub-categorized as proximal (0–50 µm), medial (50–100 µm), medial-distal (100–150 µm), and distal (150–200 µm) segments of the dendrite, depending on their distance from the soma. The total density of spines was also analyzed. Based on their morphology^23,52^, the spines were manually divided into (1) stubby, i.e., length of the protrusion was <1 µm and no neck is observed; (2) mushroom, when a clear head-like structure could be observed (maximum diameter of the head was at least 1.5 times the average length of the neck) and the total length of the protrusion was <1.5 µm; and (3) thin, i.e., the length of the protrusion was >1.5 µm or the length was between 1 and 1.5 µm and a clear head-like structure could not be distinguished.

For dendritic spine quantification using the Golgi–Cox method: Images were acquired using the Nikon Ti2 using a 100× objective (NA = 1.45). Stretches of mid-apical dendrites in CA1 pyramidal neurons were recorded with a *z*-stack size of 0.3 µm. Per animal, ten dendrites of ten different cells were quantified for the number of spines (calculated as spines per µm dendrite).

*RNAscope in-situ hybridization (ISH):* RNAscope® 2.5 HD Brown Reagent Kit (CatNo.322300), Advanced Cell Diagnostics (ACD), Hayward, CA, USA was used for the detection of EPO and EPOR mRNA. ISH was performed according to the manufacturer’s instructions. Briefly, coronal cryosections of 15 µm thickness were mounted on SuperFrostPlus Slides, dried, and stored at −80 °C. Sections were then pretreated by dropwise addition of hydrogen peroxide and incubated for 10 min at RT. Slides were immersed in boiling target retrieval buffer for 15 min, followed by incubation with protease plus for 30 min at 40 °C. Sections were then hybridized with the corresponding target probe Mm-Epo-01 (CatNo.444941) or Mm-Epor (CatNo.412351) for 2 h at 40 °C, followed by a series of amplification and washing steps. Chromogenic signal detection was performed with 3,3′-diaminobenzidine (DAB) incubation for 20 min at RT. Sections were counterstained with 50% Mayer’s hemalum (Merck) and mounted with EcoMount (BioCare Medical). Brown punctate dots in the CA1 were counted in a total of 12 hippocampi with 6 sections per animal using a light microscope (Olympus BX-50, Tokyo, Japan) equipped with a 100× oil immersion objective (NA = 1.35) and normalized to the area of the respective region (mm²). Sagittal 15 µm sections from kidney (P55) and heart (E11.5) of WT mice were used as positive controls for EPO and EPOR, respectively. According to manufacturer’s instructions, each dot represents a single molecule of mRNA in these sections. The quantification is normalized and presented as percentage of the mean dot number of groups and time points [% value = (number/mm²)/(mean dot number/mm²) × 100].

### Drop sequencing

*Tissue dissociation:* Juvenile male WT mice (P28; three mice/group to allow biological replication) were injected i.p. with placebo or EPO and sacrificed after 6 h. The hippocampi were dissected and sliced into 600 µm sections using McIlwain Tissue chopper (Cavey Laboratory Engineering Co. Ltd). The CA1 region was digested with a working solution of Papain/DNaseI in Earle’s Balanced Salt Solution, according to manufacturer’s instructions (Worthington Biochemical Corp). The samples were then incubated at 37 °C for 40 min with constant agitation before gentle manual trituration. The samples were centrifuged for 10 min at 200 × *g* at 4 °C. After discarding the Papain/DNaseI supernatant, cells were resuspended in 1 mL of sterile DMEM/F12 (Sigma) without phenol-red containing 3% fetal bovine serum (FBS; Life Technologies) and the suspension was passed through a 40 µm strainer cap (Corning) to yield a uniform single-cell suspension.

*Single-cell barcoding and library preparation:* Barcoded single cells, or STAMPs (single-cell transcriptomes attached to microparticles), and cDNA libraries were generated following the protocol^53^. Briefly, single-cell suspensions (100 cells/μl), droplet generation oil (Bio-Rad), and barcoded microparticles (ChemGenes; 120 beads/μl) were co-flowed through a FlowJEM aquapel-treated DropSeq microfluidic device (FlowJEM) and droplets were generated for 15 min. Captured RNA on the bead surface was recovered by washing the beads in saline-sodium citrate buffer and perflurooctanol solutions, and then reverse transcribed using Maxima H minus reverse transcriptase kit (ThermoFisher). Excess primer on the surface of the bead uncaptured by an RNA molecule was digested using Exonucleases I kit (ThermoFisher). A cDNA amplification PCR was performed using 10 µM SMART PCR primer and 2X Kapa HiFi HotStart ReadyMix (Kapa Biosystems) with 5000 beads per tube, and amplified for nine PCR cycles. The resulting samples were purified using AMPureXP beads (Beckman Coulter) and the quality and concentration of the cDNA was assessed using High-sensitivity DNA bioanalyzer (Agilent Technologies). Library sizes were adjusted using the Nextera Amplicon Tagmentase enzyme and DNA was amplified for 14 cycles using 10 µM New-P5-SMART PCR hybrid oligo, 10 µM Nextera Index, and the Nextera PCR mix (Nextera XT DNA Library Preparation kit; Illumina). Tagmented libraries were again purified (AMPureXP), quality controlled (high-sensitivity DNA Bioanalyzer), quantified (Qubit dsDNA HS assay kit; Life Technologies), and sequenced (Illumina Hi-seq 2500). All assays mentioned above have been performed according to manufacturer’s protocol.

*Single-cell RNA-seq processing:* Unique molecular identifier (UMI) gene counts for each group (placebo or EPO) was imported into R (v3.4.1). *Seurat* (v2.3.0) function within the R environment was used for filtering, normalization, canonical correlation analyses (CCA), unsupervised clustering, visualization, and differential expression analyses.

*Filtering and data normalization:* Cells with minimum and maximum of 1000 and 8000 genes expressed (≥1 count), respectively, and the genes that were expressed in at least three cells were retained. Cells with >40% of counts on mitochondrial genes were excluded (Supplementary Fig. 1). After filtering, there were 14,061 genes in 390 cells from placebo and 14,971 genes in 583 cells from EPO group.

Gene UMI counts for each cell were normalized via natural-log normalization of gene UMI counts divided by total UMI counts per cell and scaled by 10,000. After normalization, scaled expression (*z*-scores for each gene) for downstream analyses was calculated.

*Canonical correlation analysis:* Integration of scRNA-seq data from the two groups (placebo and EPO) was performed using CCA. Top 1000 highly variable genes from each group were used to calculate canonical correlation vectors (reduced dimension) and subsequently, first 20 vectors were aligned using dynamic time warping.

*Clustering, visualization, and differential expression:* Clustering was performed using “*FindClusters*” function with default parameters with resolution set to 1 and first 20 CCA aligned dimensions were used in the construction of the shared-nearest neighbor graph and to generate two-dimensional embeddings for data visualization using *t*SNE. Based on the visualization the glutamatergic cluster 0 and 1 were merged manually to represent a single cluster. The percentages of ‘immature glutamatergic cells’ for each mouse were: placebo 1.6%, 2.3%, and 4.3%; EPO 3.7%, 6.8%, and 7.0%. We used the “*FindAllMarkers*” function with default parameters and tested genes with a detected threshold of minimum of 25% of cells in either of the two clusters. Genes with an adjusted *p* < 0.01 were considered to be differentially expressed (for a list of cluster markers see Source Data File). A heatmap showing the top ten marker genes, i.e., differentially expressed genes per cluster is provided in the Supplementary Fig. 2.

*Cell trajectory (pseudotime) analysis:* Trajectory analysis of cells from the ‘Immature Glutamatergic’ and ‘Mature Glutamatergic1’ clusters (*n* = 502) was performed in *Monocle*2^25^. The trajectory was constructed according to the documentation of *Monocle*2. Prior to cell ordering, reclustering was performed to confirm robust detection of the immature cluster across methods, which revealed three clusters (one cluster largely corresponding to *Seurat*’s ‘Immature Glutamatergic’ cluster and two corresponding to *Seurat*’s ‘Mature Glutamatergic1’ cluster; Supplementary Fig. 3a). Subsequently, dimension reduction using the ‘DDRTree’ method was performed. Differentially expressed genes (*q* < 0.01) between the three clusters obtained by reclustering in *Monocle*2 were used as input for pseudotemporal ordering.

### Quantification and statistical analysis

All statistical analysis was performed using GraphPad Prism 5. For comparisons across multiple groups, a two-way analysis of variance (ANOVA) was used. For comparisons across two groups, an unpaired Student’s *t*-test was performed. A *p* < 0.05 is considered statistically significant. Variance was similar between compared groups for their respective experiments. All values represent mean ± SEM (standard error of the mean). All analysis and quantification were performed in a double-blinded fashion.

### Reporting summary

Further information on research design is available in the Nature Research Reporting Summary linked to this article.

## Supplementary information

Supplementary Information

Peer Review

Reporting Summary

## Data Availability

The data that support this study are available from the corresponding authors upon reasonable request. A Source Data File is provided. Raw and processed scRNA-seq data are available via the GEO with accession code GSE144444.

## Source data

Source Data File

## Code availability

Analysis scripts are accessible from github: https://github.com/AgnesSteixner/EPO_Wakhloo_et_al_NATCOMM.
