# Supplementary material for: Functional hypoxia drives neuroplasticity and neurogenesis via brain erythropoietin
Source: Nat Commun. 2020 Mar 9;11:1313. doi: 10.1038/s41467-020-15041-1 (PMC7062779; doi:10.1038/s41467-020-15041-1)
Supplement: Supplementary file 1 — Supplementary Information [file 41467_2020_15041_MOESM1_ESM.pdf]

SUPPLEMENTARY INFORMATION

Functional hypoxia drives neuroplasticity and neurogenesis via brain erythropoietin

Wakhloo et al

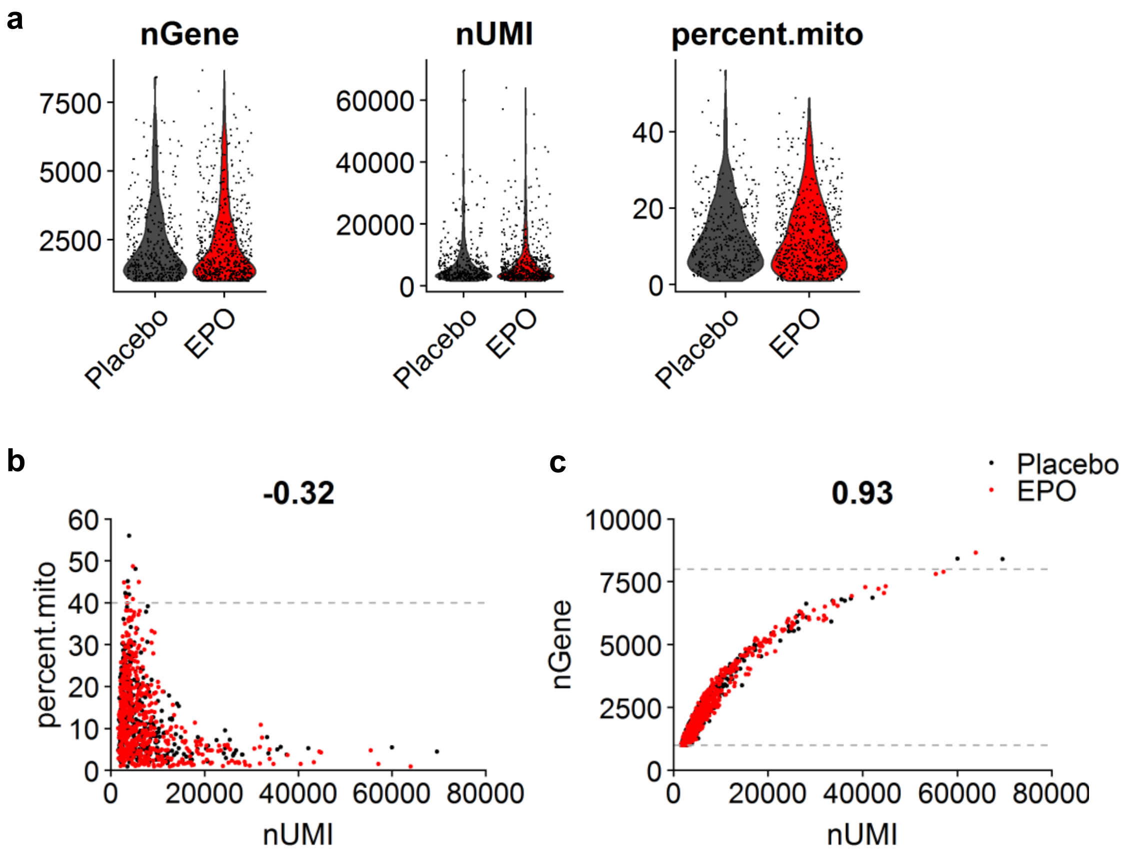

Supplementary Figure 1: **DropSeq quality control information**

Quality control information on pre-filtered (genes expressed in ≥3 cells, cells expressing ≥1000 genes) raw data from 6 male mice (P28). **(a)** Placebo and EPO treated groups are widely comparable with regard to major quality control readouts: number of genes detected (nGene), number of UMIs detected (nUMI) and percentage of mitochondrial genes expressed. **(b)** Correlation of percentage of mitochondrial genes expressed (percent.mito) and number of UMIs detected (nUMI), colored by treatment group. Cells above the dashed grey line were discarded before downstream analysis. **(c)** Correlation between number of genes (nGene) and number of UMIs detected (nUMI), colored by treatment group. Cells outside the upper and lower dashed grey lines were discarded before downstream analysis.

Wakhloo et al Supplementary Figure 2

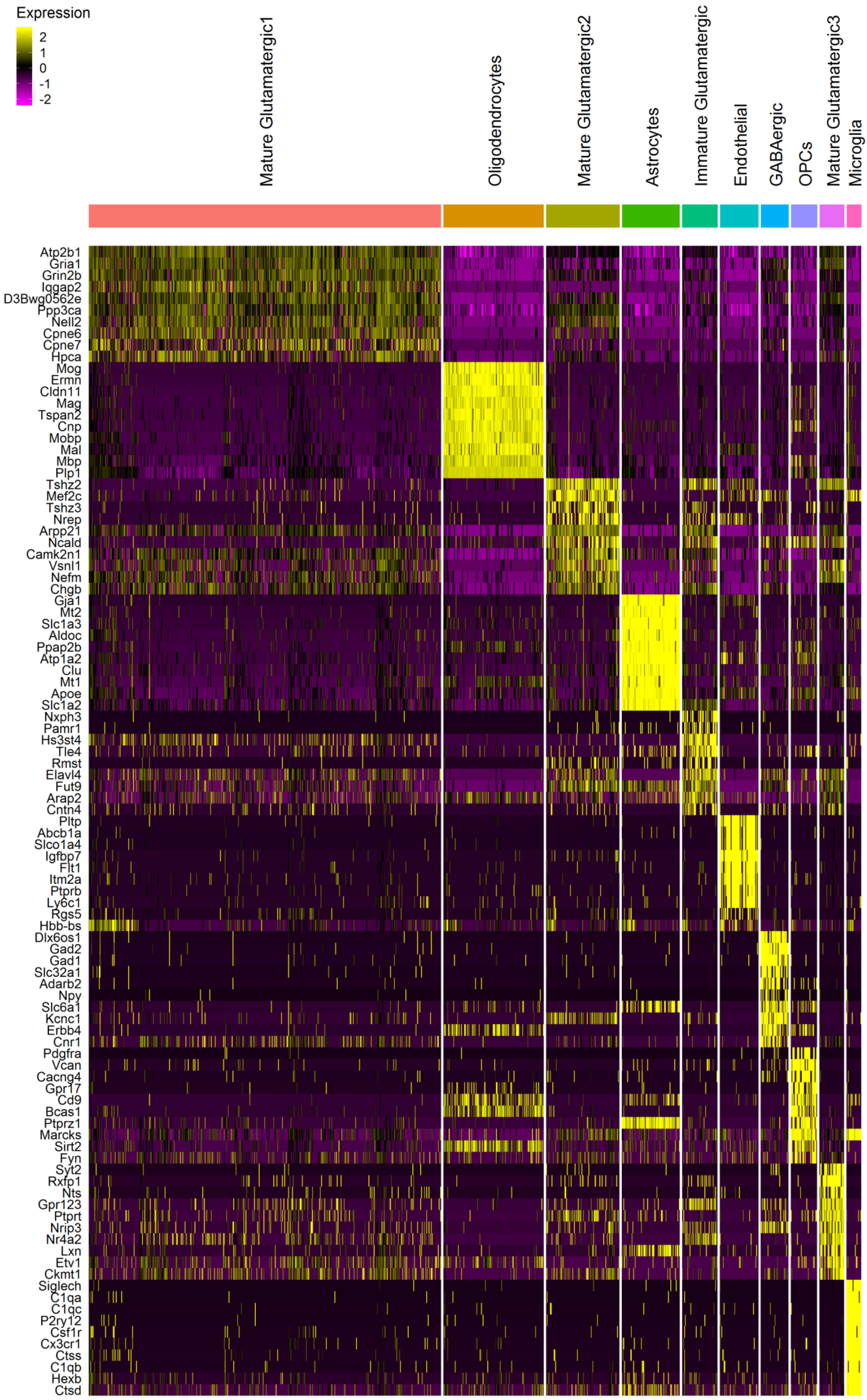

Supplementary Figure 2: **Heatmap of top 10 marker genes (ordered by average log fold-change) per cluster.**

Wakhloo et al Supplementary Figure 3

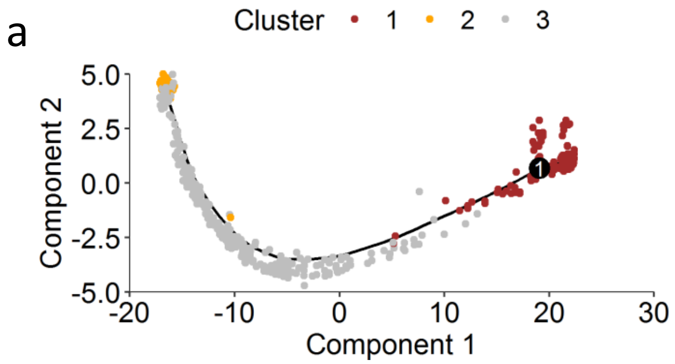

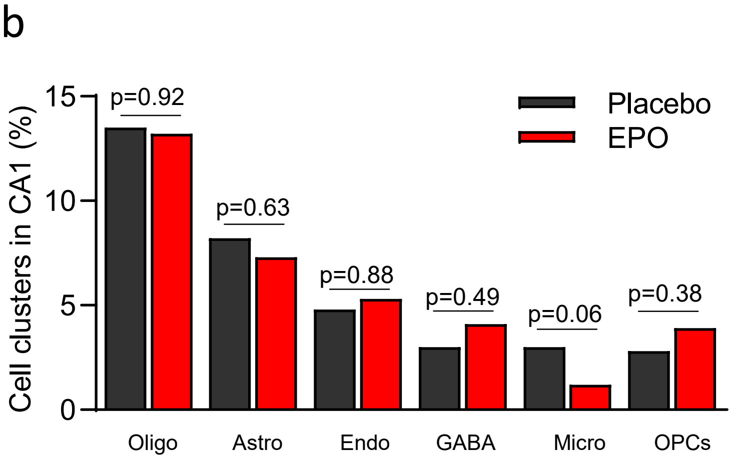

Legend
Oligo: Oligodendrocytes
Astro: Astrocytes
Endo: Endothelial cells
GABA: GABAergic neurons
Micro: Microglia
OPCs: Oligodendrocyte percursor cells

c

	% positive cells							
	Tbr1	Dcx	Tle4	Any	Triple+	Tbr1+Dcx	Tbr1+Tle4	Tle4+Dcx
**Immature (pseudotime >15)**	60	30	100	100	20	20	60	30
**Immature (pseudotime < 15)**	33.3	33.3	69.4	88.9	8.3	13.9	25	16.7
**Mature**	9.6	8.6	18.6	30.7	0.7	1.8	3.3	1.8

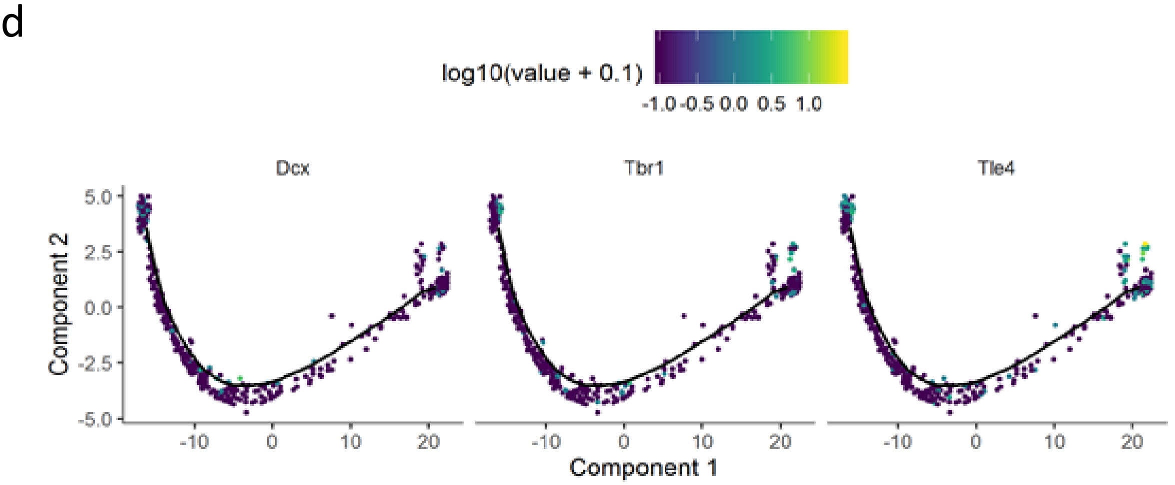

e

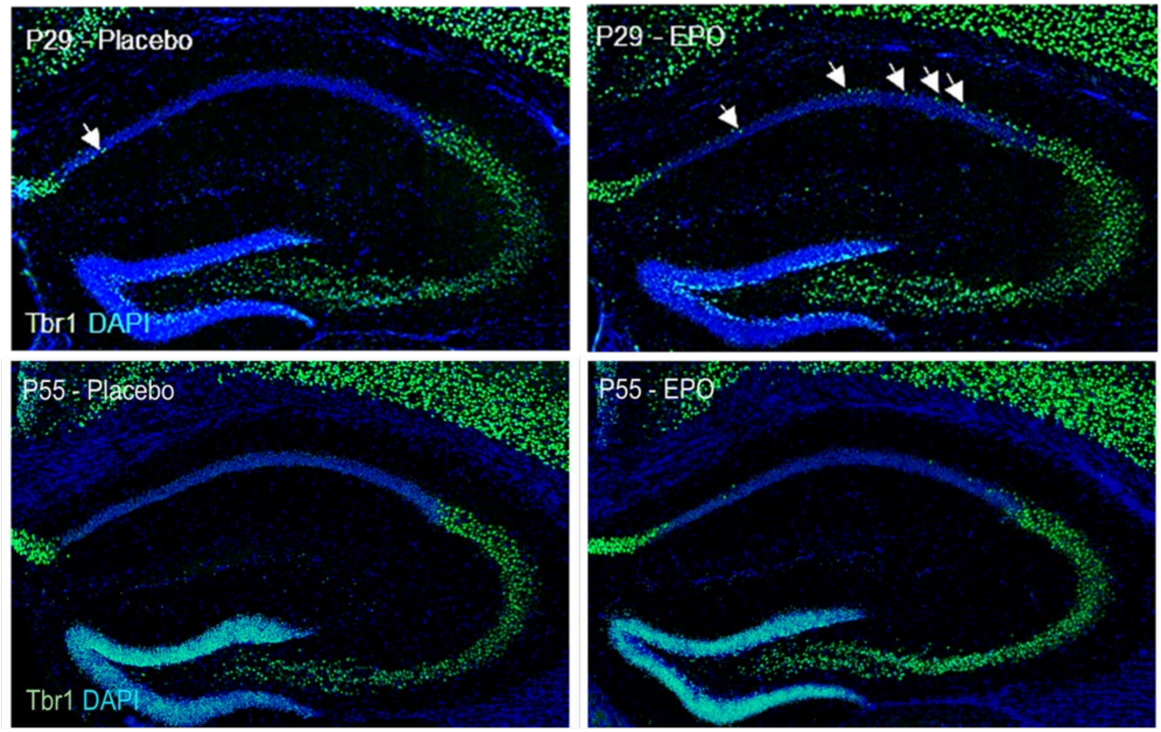

Supplementary Figure 3: **(a) Characterization of the immature glutamatergic cluster by trajectory analysis.** Trajectory colored by clusters as identified by *Monocle2*. Cluster 2 (yellow) widely corresponds to the 'Immature Glutamatergic' cluster defined by Seurat. Clusters 1 (brown) and 3 (grey) largely correspond to the 'Mature Glutamatergic1' cluster (Figure 2g). **(b) Percentage of cells in the respective non-glutamatergic cell clusters** per treatment condition (EPO: n=583, placebo: n=390); 2-tailed Fisher's exact test. **(c) Percentage of cells expressing immature cluster markers.** The table-figure shows the percentage of cells included in the trajectory analysis (split by pseudotime, i.e. high vs. low, and cluster identity) that are positive for 3 characteristic markers taken as examples of the immature cluster. Percentage of cells expressing each marker (and their different combinations) within 3 cell groups, i.e. immature with high pseudotime, immature with low pseudotime and mature cells, are provided. Note that marker expression of immature cells with high pseudotime resembles more closely that of immature cells with low pseudotime than that of mature cells, suggesting an immature identity of these cells despite of their high pseudotime. In particular, Dcx - a well-established marker of immature neurons - is detected in 30% of immature neurons with pseudotime >15 and 33% of immature neurons with pseudotime <15, but only in 8.6% of mature neurons, providing additional support for the immature identity of cells. **(d) Immature cluster marker expression along the trajectory.** Expression of 3 selected prominent markers of the immature cluster along the trajectory, showing that the population of immature neurons with pseudotime >15 clearly expresses these 3 markers highly similar to the remaining immature neurons. **(e) Tbr1 expression in the hippocampus.** IHC staining for Tbr1 at P29 and P55 shows that in CA1, Tbr1 upon EPO is strongly detected at P29 but rarely at P55. Tbr1+ cells in dentate gyrus serve as positive control for both time-points.

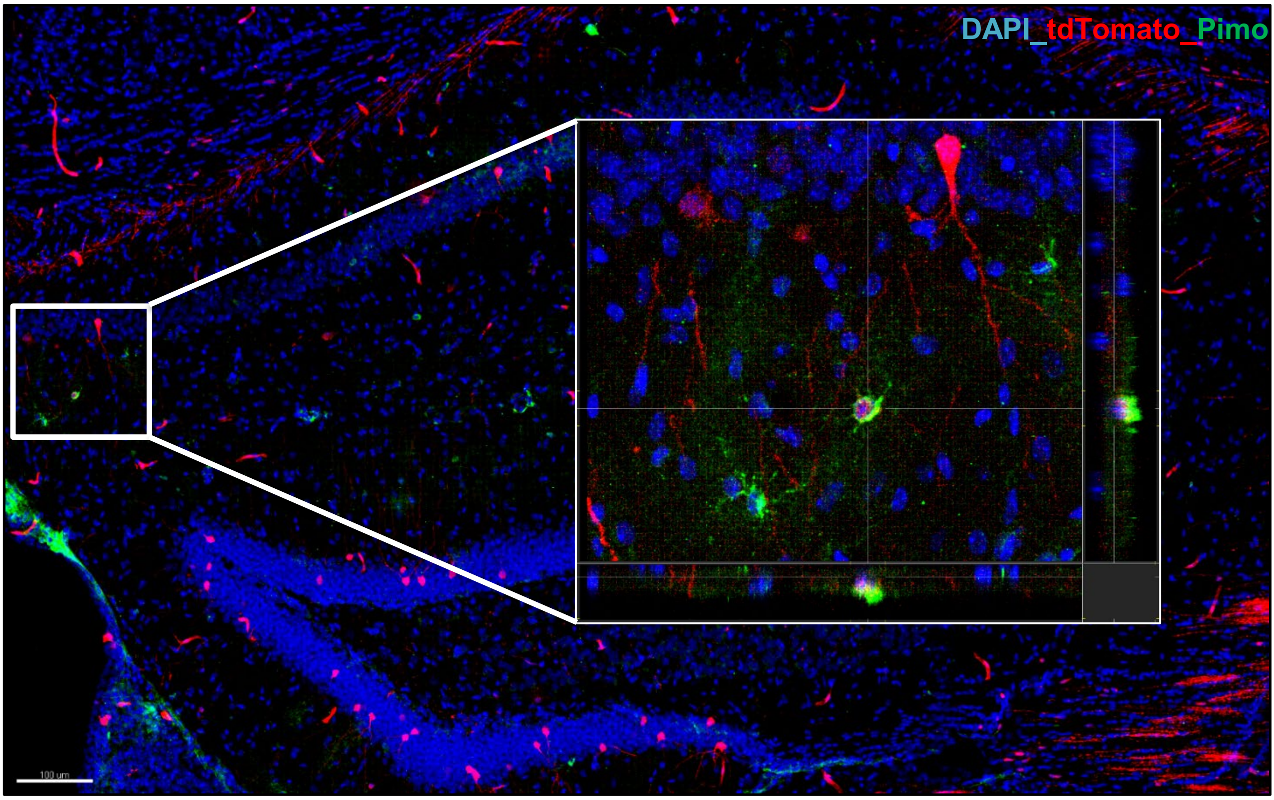

***Supplementary Figure 4:* Validation of hypoxia by pimonidazole staining**

Hypoxic cells were validated with pimonidazole staining (according to *Sato et al 2011*) in CAG-CreERT2-ODD::R26R-tdTomato mice. Mice received a single tamoxifen injection (100mg/kg body weight), followed after 16 hours by pimonidazole administration, and were sacrificed 90min later. Double-labeled hypoxic cells occur typically dispersed (compare *Kimura et al 2015*). Scale bar represents 100µm in the overview image.

Pimonidazole (Hypoxyprobe-1 Mab1) from NPI (Belmont, MA USA) is used to detect hypoxia in cells and tissue. Pimonidazole (60mg/kg body weight) solution is injected intravenously (tail vein) and staining performed according to manufacturer‘s instructions.

Wakhloo et al Supplementary Figure 5

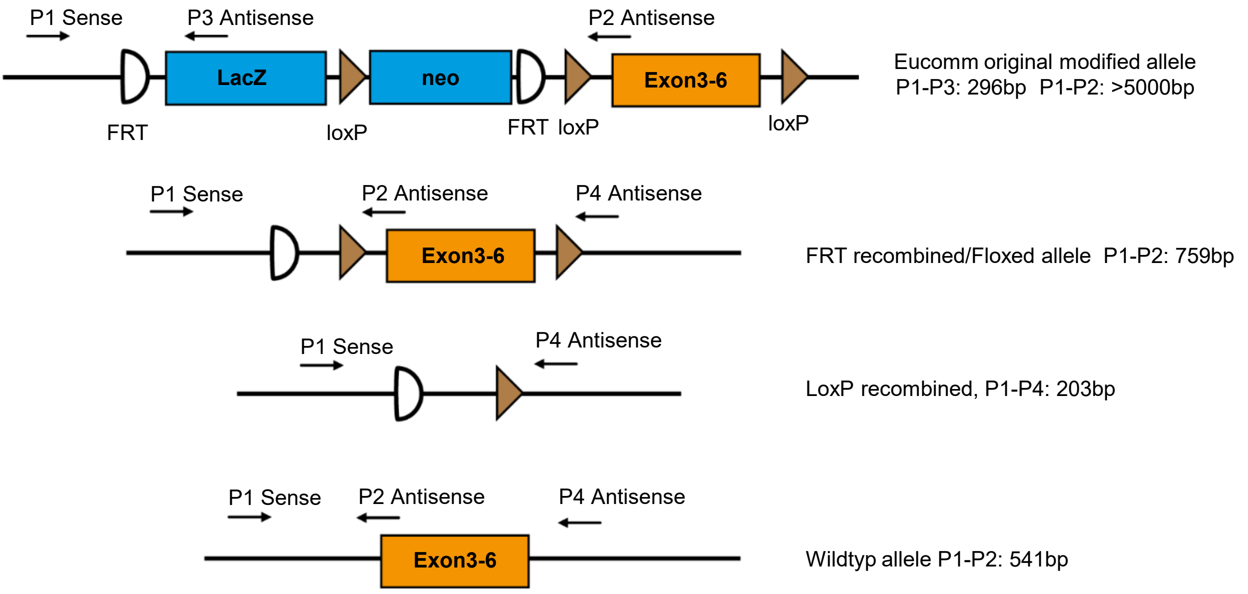

P1: 29825 5‘-GTCCTCAGGACTGGGTGGC-3‘
P2: 29826 5‘-CATCGATGTCGCTCCCAT-3‘
P3: 15048 5‘-CAACGGGTTCTTCTGTTAGTCC-3‘
P4: 32002 5‘-GAGCTCAGACCATAACTTCG-3‘

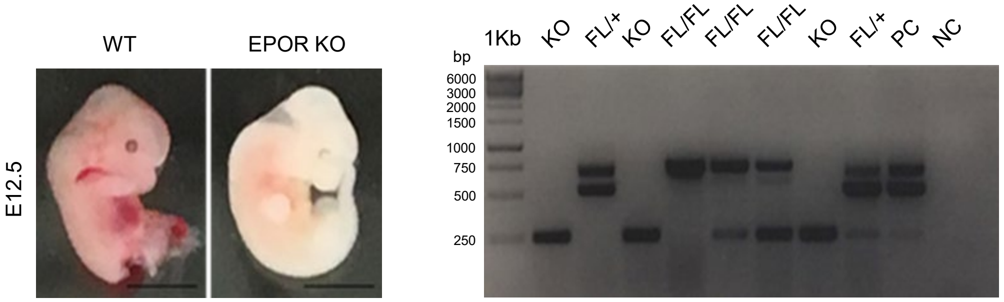

Supplementary Figure 5: **Description of pyramidal neuronal EPOR KO**

To generate the specific conditional EPOR KO in pyramidal neurons (NexCre::EPORflox/flox), we had to first validate whether the generated EPOR KO mice are indeed functional knockouts. Therefore cross breeding of the female EPORflox/flox mice with male mice homozygous for the Cre-recombinase gene (under control of the ubiquitous adenovirus EIIA-promoter), was used to obtain deleter mice. EIIA-regulated Cre-recombinase was expressed in pre-implantation embryos leading to site specific deletion of LoxP flanked (fl) sequence in all tissues including germ cells. Interbreeding of first generation progenies resulted in efficient germline transmission of the deletion to subsequent generations. The presence of deleted alleles was determined by PCR-based genotyping using specific primers for EPOR (listed below) and also further confirmed using EIIA-cre primers. We were able to observe a global EPOR KO at E12.5, validating functionality of generated EPOR KO mice. P1-4 (primer 1-4); E12.5 (embryonic day 12.5); PC (positive control); NC (negative control).

Supplementary References

Sato, Y., Endo, H., Okuyama, H., Takeda, T., Iwahashi, H., Imagawa, A., Yamagata, K., Shimomura, I. & Inoue, M. Cellular hypoxia of pancreatic β-cells due to high levels of oxygen consumption for insulin secretion in vitro. *Journal of Biological Chemistry* **286**, 12524-12532 (2011).

Kimura, W., Xiao, F., Canseco, D. C., Muralidhar, S., Thet, S., Zhang, H. M., Abderrahman, Y., Chen, R., Garcia, J. A., Shelton, J. M., Richardson, J., Abderrahman, A., Aroumougame, A., Liang, H., Xing, C., Lu, Z., Zhang, C. & Sadek, H. Hypoxia fate mapping identifies cycling cardiomyocytes in the adult heart. *Nature* **523**, 226 (2015).
