# Supplementary material for: Functional hypoxia drives neuroplasticity and neurogenesis via brain erythropoietin
Source: Nat Commun. 2020 Mar 9;11:1313. doi: 10.1038/s41467-020-15041-1 (PMC7062779; doi:10.1038/s41467-020-15041-1)
Supplement: Supplementary file 2 — Peer Review [file 41467_2020_15041_MOESM2_ESM.pdf]

Editorial Note: This manuscript has been previously reviewed at another journal that is not operating a transparent peer review scheme. This document only contains reviewer comments and rebuttal letters for versions considered at *Nature Communications.*

Reviewers' comments:

Reviewer #1 (Remarks to the Author):

The authors have maintained focus on a lineage speccific question in neurons despite it being highly related to prior findings in oligiogendeocytes by saying this is not their question. Well, perhaps their question is not the best question! The narrow framing seems to have missed an opportunity to make a more significant biological advnace.

Having said this, I am quite impressed with overall improvements to the paper in the current version and the careful attention to all reviewers points. I recommend publication without major revisions and have no additional comments to make except that the discussion should expand on the point that neuron-glial interactions are an impportnat area for future study.

Reviewer #2 (Remarks to the Author):

The manuscript by Wakhloo et al. provides two major findings: (1) Epo-mediated de-novo generation of pyramidal neurons without prior divisions, but transformation and (2) that learning induces transient ischemia that in turn activates Epo/EpoR axis in pyramidal neurons that in a paracrine-mode ultimately lead to de-novo generation of neurons in CA1 layer of the hippocampus.
To substantiate the first finding they used single cell transcriptomics to identify immature stages of these de-novo generated neurons. Unfortunately, they only found very few cells that would fulfill this criteria in the EPO-treated mice as compared to Controls in the tSNE plot (Seurat). These few cells could not entirely be validated using pseudotime analysis. A number of cells from Seurat's immature glutamatergic cluster are located at the very end of the pseudotime trajectory (Fig. 2g), indicating that they may actually be mature. The true identity of these cells may now be investigated in various laborious ways, but ultimately the easiest and most convincing way to deal with them is to simply repeat Fisher's exact test without them. Since this requires minimal effort, it is surprising that the authors did not fulfill this basic request. I accept that the authors want to refrain from manually adjusting cluster assignments e.g. in the t-SNE figure. The point of repeating Fisher's test is merely to demonstrate that the test results do not hinge on this small set of "shaky" cells. The test on Monocle's "Cluster 2" indeed suggests that this is not the case, however it is easier for reviewers and readers alike to comprehend the Seurat analysis which has been laid out in greater detail in the main manuscript, as opposed to the Monocle analysis where it is not as clear what the different clusters represent. I would like to re-state my request to repeat Fisher's exact test after excluding the immature cells with >15 pseudotime.Therefore, we asked to re-assign the cells qualifying as mature neurons in pseudotime and assign in the t-SNE plot and to repeat the fisher test with corrected numbers. This is crucial, as t-SNE is a tool offering a 2D visualizations of cells likely exhibiting a similar transcriptome where cells at the border can be wrongly assigned.
The authors further based their reluctance to follow my suggestion on the fact that these wrongly assigned cells do express the markers of immature cells tbr1, tle4 and Dcx. If this is the case, I would like to know how many of the cells assign as mature cell exhibit expression of these markers and vice versa. Can the authors use the single expression of these markers to distinguish the different maturation stages on the transcriptome? In the link to available transcriptome data from the Linnarsson's lab (even provided as supporting information in the response to the reviewers page 8) all three markers in isolation can be found in some mature granule neurons. In addition, the lack of biological replicates for the cells used in the tSNE plot allows only nominal p-values. So it remains unclear whether this is relevant at all.
Regarding the tbr1 protein: in the panel d and f in page 8 of the response to the reviewer, the Tbr1

positive cells within the dentate gyrus, which should serve as a positive control are only detected in the P29-EPO image. Are these pictures taken at the same laser intensity?

January 15, 2020

Hannelore Ehrenreich and Klaus-Armin Nave
Max Planck Institute of Experimental Medicine

Wakhloo et al *Functional hypoxia drives neuroplasticity and neurogenesis via brain erythropoietin*.

Point-to-Point Response and RE-RE-REVISION of NCOMMS-19-34458-T

- **Red: Reviewer's Questions (3nd round)**
- Black: Our Point-to-Point Response
- New data / text in revised paper

Reviewer #1 (Remarks to the Author):

The authors have maintained focus on a lineage speccific question in neurons despite it being highly related to prior findings in oligiogendeocytes by saying this is not their question. Well, perhaps their question is not the best question! The narrow framing seems to have missed an opportunity to make a more significant biological advnace.

We thank the reviewer for the feedback and for taking the time to review our manuscript once more.

Having said this, I am quite impressed with overall improvements to the paper in the current version and the careful attention to all reviewers points. I recommend publication without major revisions and have no additional comments to make except that the discussion should expand on the point that neuron-glial interactions are an impportnat area for future study.

We have now included in the discussion the point that neuron-glial interactions are an important area for future study, page 9.

Reviewer #2 (Remarks to the Author):

The manuscript by Wakhloo et al. provides two major findings: (1) Epo-mediated de-novo generation of pyramidal neurons without prior divisions, but transformation and (2) that learning induces transient ischemia that in turn activates Epo/EpoR axis in pyramidal neurons that in a paracrine-mode ultimately lead to de-novo generation of neurons in CA1 layer of the hippocampus.

We thank the reviewer for taking again the time to read our paper as well as our extensive response, and for acknowledging our major findings in this final round of review.

To substantiate the first finding they used single cell transcriptomics to identify immature stages of these de-novo generated neurons. Unfortunately, they only found very few cells that would fulfill this criteria in the EPO-treated mice as compared to Controls in the tSNE plot (Seurat). These few cells could not entirely be validated using pseudotime analysis. A number of cells from Seurat's immature glutamatergic cluster are located at the very end of the pseudotime trajectory (Fig. 2g), indicating that they may actually be mature. The true identity of these cells may now be investigated in various laborious ways, but ultimately the easiest and most convincing way to deal with them is to simply repeat Fisher's exact test without them. Since this requires minimal effort, it is surprising that the

authors did not fulfill this basic request. I accept that the authors want to refrain from manually adjusting cluster assignments e.g. in the t-SNE figure. The point of repeating Fisher's test is merely to demonstrate that the test results do not hinge on this small set of "shaky" cells. The test on Monocle's "Cluster 2" indeed suggests that this is not the case, however it is easier for reviewers and readers alike to comprehend the Seurat analysis which has been laid out in greater detail in the main manuscript, as opposed to the Monocle analysis where it is not as clear what the different clusters represent. I would like to re-state my request to repeat Fisher's exact test after excluding the immature cells with >15 pseudotime. Therefore, we asked to re-assign the cells qualifying as mature neurons in pseudotime and assign in the t-SNE plot and to repeat the fisher test with corrected numbers. This is crucial, as t-SNE is a tool offering a 2D visualizations of cells likely exhibiting a similar transcriptome where cells at the border can be wrongly assigned.

We now present the requested Fisher's exact test (one-sided post hoc test) after excluding the immature cells with >15 pseudotime: Even after exclusion of the 10 cells with pseudotime >15, the increase in immature cells is still significant at p=0.039. If the immature cells with high pseudotime (>15) are re-assigned to the mature glutamatergic cluster, we still obtain p=0.041. This is now included also in the manuscript, page 4.

However, as shown in the table below, ***provided for the reviewer***, the marker expression of the immature cells with high pseudotime is much more similar to that of the immature cells with low pseudotime than to the mature cells, pointing to an immature identity of these cells (particularly the Dcx expression should remove any doubt – see also below). In addition, we would like to stress again, that we also provided immunostaining for Tbr1 and Tle4, which nicely confirmed on protein level the increase in immature neurons carrying these two markers – see Figure 2e.

The authors further based their reluctance to follow my suggestion on the fact that these wrongly assigned cells do express the markers of immature cells tbr1, tle4 and Dcx. If this is the case, I would like to know how many of the cells assign as mature cell exhibit expression of these markers and vice versa. Can the authors use the single expression of these markers to distinguish the different maturation stages on the transcriptome? In the link to available transcriptome data from the Linnarsson's lab (even provided as supporting information in the response to the reviewers page 8) all three markers in isolation can be found in some mature granule neurons.

The table below – ***presented for the reviewer*** - shows the percentages of cells positive for each marker (and their different combinations) for each cell group, i.e. immature with high pseudotime, immature with low pseudotime and mature cells. Furthermore, we provide a graph showing the expression of the 3 markers along the trajectory. We hope to hereby alleviate the reviewer's concerns with regard to the questioned immature identity of cells, since these data show that the population of immature neurons with pseudotime >15 clearly expresses the 3 markers and is highly similar to the remaining immature neurons.

As shown in the table, the immature cluster (with pseudotime greater or smaller 15) has a considerably higher percentage of cells expressing Tbr1, Dcx and Tle4 as compared to the mature cluster. Therefore, these markers – in isolation or together – appear to be characteristic for this cluster (again not unexpected, due to them being initially identified as marker genes of this cluster via differential gene expression analysis). In this sense, these 3 markers allow to differentiate between immature and mature neurons and therefore differentiation stages, e.g. triple-positive cells are very rare (<1%) in the mature cluster but are found in 8-20% of immature cells. Regarding

the question if the immature cells with Monocle's pseudotime >15 are indeed immature, we hope to finally convince the reviewer with the fact that Dcx is expressed in 30% of immature neurons with pseudotime >15 (highly similar to 33% in immature neurons with pseudotime <15), but only in 8.6% of mature neurons. This should provide additional support for the immature identity of the cells in question, since Dcx is a well-established marker of immature neurons and its expression known to subside upon maturation.

With regard to the Linnarson data set, we would like to point the reviewer's attention to dataset C (http://linnarssonlab.org/dentate/), where **immature** pyramidal neurons (in DG) are shown to express all these 3 markers (which are more relevant for us as compared to the mature granule neurons mentioned by the reviewer). Unfortunately, these datasets do not contain mature pyramidal neurons of CA1 that would allow for a direct comparison with the mature neurons in our data.

	**% positive cells**							
	**Tbr1**	**Dcx**	**Tle4**	**Any**	**Triple+**	**Tbr1+Dcx**	**Tbr1+Tle4**	**Tle4+Dcx**
**Immature (pseudotime >15)**	60	30	100	100	20	20	60	30
**Immature (pseudotime < 15)**	33.3	33.3	69.4	88.9	8.3	13.9	25	16.7
**Mature**	9.6	8.6	18.6	30.7	0.7	1.8	3.3	1.8

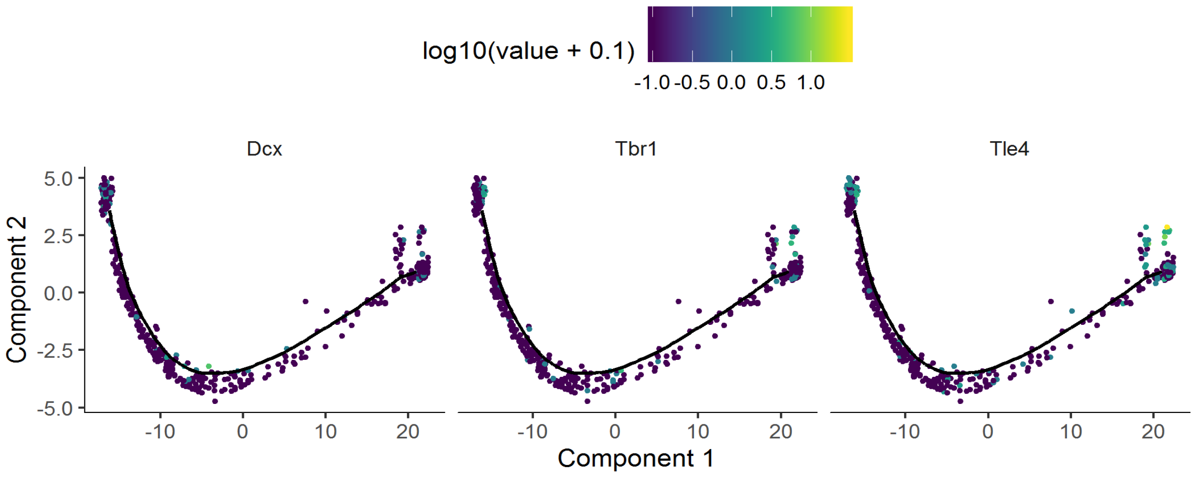

In addition, the lack of biological replicates for the cells used in the tSNE plot allows only nominal p-values. So it remains unclear whether this is relevant at all.

We are sorry that our **biological replicates** were obviously not clearly enough mentioned which has led to misunderstandings by this reviewer. This has now been corrected and made even clearer in the revised manuscript, pages 12, 22, 24.

We analyzed in total 3 mice per treatment group, i.e. 2 biological replicates each, always shown together in the graphical representation. In order to exclude that the observed increase is driven by a deviation of cell numbers in one mouse, we had already provided the percentage of immature cells for each mouse separately in the methods section (page 24), which shows that the tendency of an increase in

immature cells under EPO is present in all mice. In addition, we provided immunostaining results for Tbr1 and Tle4 (figure 2e) as mentioned above, confirming the observed increase also on the protein level.

Regarding the tbr1 protein: in the panel d and f in page 8 of the response to the reviewer, the Tbr1 positive cells within the dentate gyrus, which should serve as a positive control are only detected in the P29-EPO image. Are these pictures taken at the same laser intensity?

In fact, the Tbr1 positive cells within the dentate gyrus, serving as a positive control are also detected in the P55-EPO image. This P55 image was only presented for the reviewer's interest. However, the reviewer is correct, that the laser intensity at which the pictures were taken was different (P29 – 191ms versus P55 – 200ms). We now prepared and provide below, again for the reviewer's interest, the P29 images next to the P55 images taken at the same laser intensity (200ms).

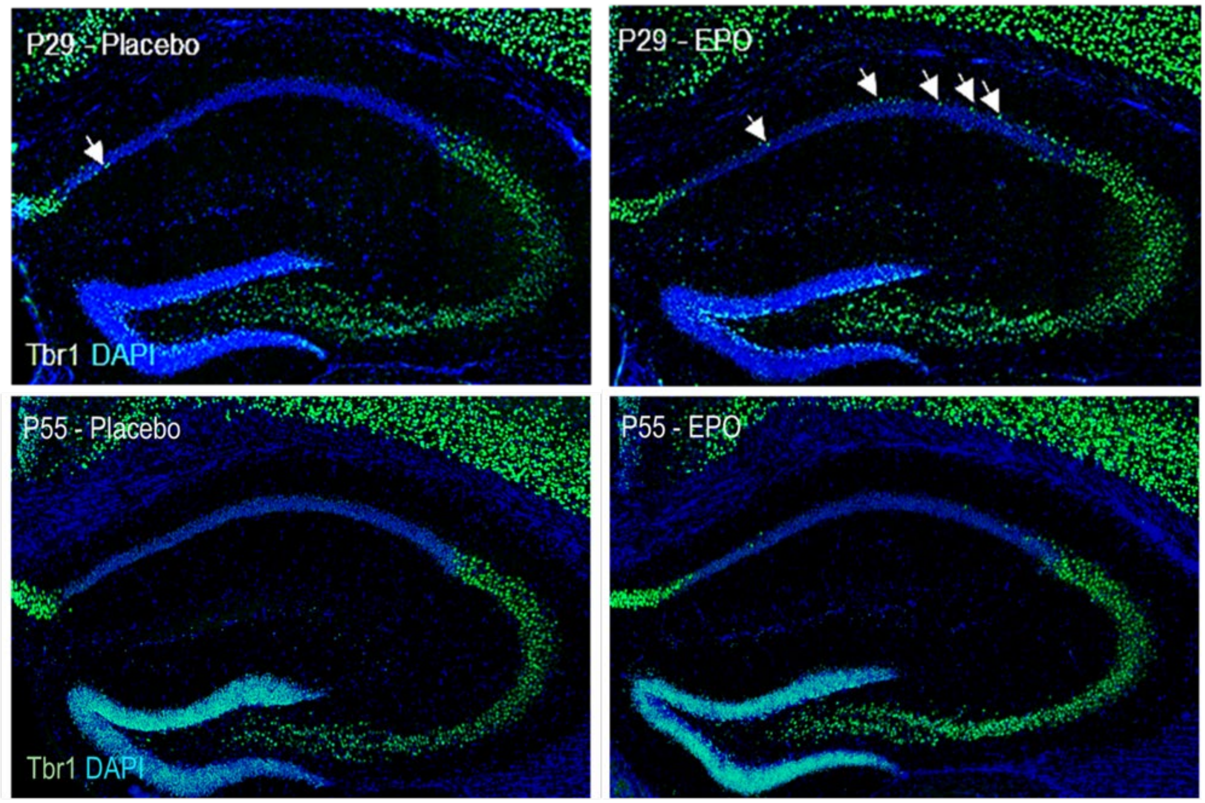

REVIEWERS' COMMENTS:

Reviewer #3 (Remarks to the Author):

The authors have addressed my previous concern and improved the statistics and image acquisition. Having said that, I still think that one analyses the whole transcriptome of an individual cell not to draw conclusions on cell type based on expression of three markers, that are not even detected together in every cell. The more appropriate way of doing it, is looking at the distances between cells which is done by pseudotime or Louvain algorithm.
Nevertheless the author want to based their claim on markers, and for this I strongly recommend showing the data provided for the reviewer in the manuscript. It should be added to the supplementary material. Also the newly acquired images.

January 30, 2020

Hannelore Ehrenreich and Klaus-Armin Nave, Max Planck Institute of Experimental Medicine

Point-to-Point Response & RE-RE-RE-REVISION NCOMMS-19-34458A

- **Red: Reviewer's Questions (4th round)**
- Black: Our Point-to-Point Response
- New data / text in revised paper

Reviewer #3 (Remarks to the Author):

The authors have addressed my previous concern and improved the statistics and image acquisition.

We thank the reviewer for taking the time to review our manuscript once more.

Having said that, I still think that one analyses the whole transcriptome of an individual cell not to draw conclusions on cell type based on expression of three markers, that are not even detected together in every cell. The more appropriate way of doing it, is looking at the distances between cells which is done by pseudotime or Louvain algorithm.

We would like to stress that the definition of all cell types, including the immature cluster, is certainly not based on 3 markers but derived from machine learning on a big portion of the cellular transcriptome by using highly variable genes. The 2 markers that we used for IHC validation were selected as examples and because of the availability of valid antibodies. As shown in the Source Data File and mentioned in the manuscript (pages 4-5), there is a large number of other transcripts that show relatively high specificity (pct.1 = percentage of immature cells expressing this marker, pct.2= percentage of all other cells expressing this marker) for cells in this cluster, e.g.

Gene	p_val	avg_logFC	pct.1	pct.2	p_val_adj
Nxph3	1,22E-64	1,199284	0,522	0,017	1,87E-60
Grik3	1,09E-49	0,855689	0,565	0,036	1,67E-45
Cntnap4	2,09E-44	0,902547	0,457	0,024	3,2E-40
Pamr1	1,04E-40	1,056429	0,435	0,025	1,6E-36
Cbln2	1,56E-37	0,976859	0,435	0,028	2,39E-33
Hs3st4	1,1E-30	1,472928	0,826	0,205	1,69E-26
Cwh43	4,67E-27	0,676047	0,326	0,023	7,15E-23
Filip1l	3,39E-25	0,96381	0,478	0,06	5,2E-21
Cntn6	6,09E-25	0,708773	0,304	0,022	9,32E-21
Rmst	1,14E-24	1,065678	0,435	0,051	1,74E-20
Etl4	1,07E-23	0,871355	0,609	0,112	1,65E-19

Since we already showed the pseudotime and Lovain clustering, including all the tests and exclusions suggested by the reviewer (which all basically confirmed our assumptions and claims), we are not sure, which additional information we should provide. In case the editor has any further suggestions which data needs to be shown or added, we are happy to do so. For now, we have included the data, originally provided for the reviewer's interest also in the supplementary section (Supplementary Figure 3 c-d), as requested.

Nevertheless the author want to based their claim on markers, and for this I strongly recommend showing the data provided for the reviewer in the manuscript. It should be added to the supplementary material. Also the newly acquired images.

The data provided for the reviewer have now been added to the supplementary material (Supplementary Figure 3 c-d). In addition, the newly acquired images of Tbr1 are integrated into this figure.
